# Genome-Wide Linkage and Association Analysis Identifies Major Gene Loci for Guttural Pouch Tympany in Arabian and German Warmblood Horses

**DOI:** 10.1371/journal.pone.0041640

**Published:** 2012-07-27

**Authors:** Julia Metzger, Bernhard Ohnesorge, Ottmar Distl

**Affiliations:** 1 Institute for Animal Breeding and Genetics, University of Veterinary Medicine Hannover, Hannover, Germany; 2 Clinic for Horses, University of Veterinary Medicine Hannover, Hannover, Germany; Institut Jacques Monod, France

## Abstract

Equine guttural pouch tympany (GPT) is a hereditary condition affecting foals in their first months of life. Complex segregation analyses in Arabian and German warmblood horses showed the involvement of a major gene as very likely. Genome-wide linkage and association analyses including a high density marker set of single nucleotide polymorphisms (SNPs) were performed to map the genomic region harbouring the potential major gene for GPT. A total of 85 Arabian and 373 German warmblood horses were genotyped on the Illumina equine SNP50 beadchip. Non-parametric multipoint linkage analyses showed genome-wide significance on horse chromosomes (ECA) 3 for German warmblood at 16–26 Mb and 34–55 Mb and for Arabian on ECA15 at 64–65 Mb. Genome-wide association analyses confirmed the linked regions for both breeds. In Arabian, genome-wide association was detected at 64 Mb within the region with the highest linkage peak on ECA15. For German warmblood, signals for genome-wide association were close to the peak region of linkage at 52 Mb on ECA3. The odds ratio for the SNP with the highest genome-wide association was 0.12 for the Arabian. In conclusion, the refinement of the regions with the Illumina equine SNP50 beadchip is an important step to unravel the responsible mutations for GPT.

## Introduction

The guttural pouches are air filled *diverticula* of the auditory tube in the caudal portion of the horse’s head. Among all mammalian species, horses developed the largest guttural pouch. Other mammalian families possessing a diverticulum of the auditory tube include *Tapiridae, Rhinocerotidae, Hydracoidea,* some species of *microchiopterans* and *Heteromys anomales*.

The equine guttural pouches may have an important function during heavy exercise for brain cooling [1]. During times of high thermal stress pharyngeal orifices open to allow ventilation of the guttural pouches. Guttural pouch tympany (GPT) is characterized by an abnormal distention of one or both guttural pouches, sometimes accompanied with some fluid accumulation. Signs of GPT become evident in the first weeks of life. Fillies are two- to fourfold more often GPT-affected than colts. With progressive inflammation of the guttural pouches, GPT causes dyspnoea, pharyngeal stridores, dysphagia and aspiration pneumonia. Without appropriate surgical treatment, this condition is life-threatening [2]. The air in the guttural pouches gets trapped because the mucosal flap at the pharyngeal orifice acts as a one-way valve, allowing the air to enter but impeding the air flow into the nasopharynx. The aetiology of this condition is unknown, but several theories are proposed, like an over-sized mucosal flap, neuromuscular dysfunction and inflammations from an upper airway infection. Nevertheless, in most cases no anatomic abnormalities have been seen in affected foals during the endoscopic examination [3].

The majority of cases for GPT had been reported for Arabian, English thoroughbreds, Standardbreds, Appaloosas, Quarter, warmblood, American saddle and Paint horse [3], [4]. A recessive major gene had been shown for German warmblood horses and a polygenic or mixed monogenic-polygenic inheritance was most likely for Arabian horses [3], [5], [6]. A whole genome scan using 257 microsatellites for Arabian and German warmblood horses showed a genome-wide significant QTL on ECA15 at 65–87 Mb for Arabian colts [7]. Haplotype analysis identified haplotypes including three microsatellites at 74–78 Mb on ECA15 as being significantly associated with GPT for Arabian colts. The objective of this study was to perform a genome-wide linkage and association study in Arabian and German warmblood horses using the Illumina equine SNP50 beadchip.

## Results

### Linkage Mapping

A genome-wide mapping strategy using highly dense single nucleotide polymorphism (SNP) sets for linkage and association analyses may be the most appropriate approach to identify the target genomic regions and trait-associated SNPs. This study shows the effectiveness of using Illumina equine SNP50 beadchip with about 55,000 evenly spaced SNPs for both, linkage and association analyses, when inheritance and pedigrees are complex.

**Figure 1:**
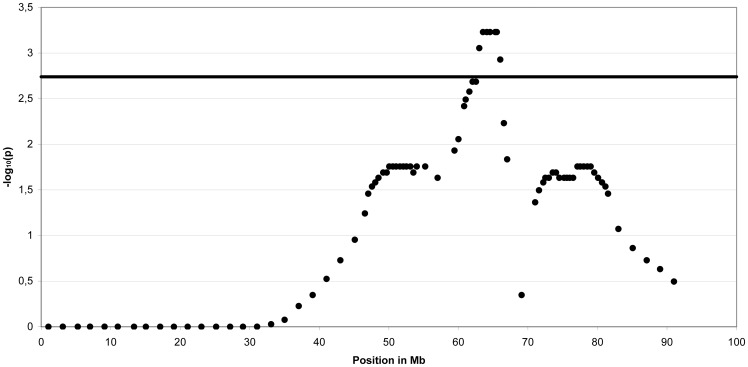
Non-parametric multipoint linkage analysis for guttural pouch tympany in Arabian. Plot of **−**log_10_ chromosome-wide significant P-values (−log_10_P) for horse chromosome (ECA) 15 using non-parametric multipoint analysis in five Arabian families. P-values are genome-wide significant above the −log_10_P threshold value of 2.74. The maximum of the curve is at 64–65 Mb and represented by four SNPs.

**Figure 2:**
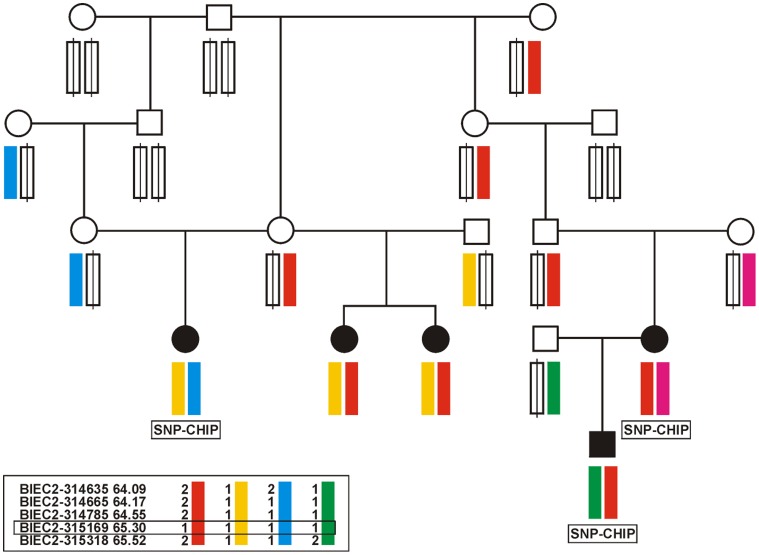
SNP-haplotypes of the Arabian family 1 in the linkage region for guttural pouch tympany (GPT) at 64–65 Mb on ECA15. Four different haplotypes are shown for five GPT-affected horses. All GPT-affected horses of the Arabian family 1 are homozygous at the BIEC2-315169 SNP at 65.30 Mb.

The multipoint non-parametric linkage analyses using 4473 SNPs with an average distance of 500 kb revealed the highest peak on ECA15 for Arabian with genome-wide significance (Figure 1) and the next highest peak on ECA3. An increased marker density for these two chromosomes using 248 SNPs with an average distance of 91 kb (ECA3) and 78 kb (ECA15) affirmed genome-wide significant linkage on ECA15 and did not confirm a genome-wide significance on ECA3 (Figure S1). For the Arabian, the whole region with chromosome-wide significant linkage on ECA15 extended across 34 Mb at 47–81 Mb (Table S1). The highest genome-wide significant peaks were detected at 64–65 Mb and this peak-region marked the most common haplotypes for affected foals. A common homozygous haplotype could not be identified in the affected foals (Figure 2).

**Figure 3:**
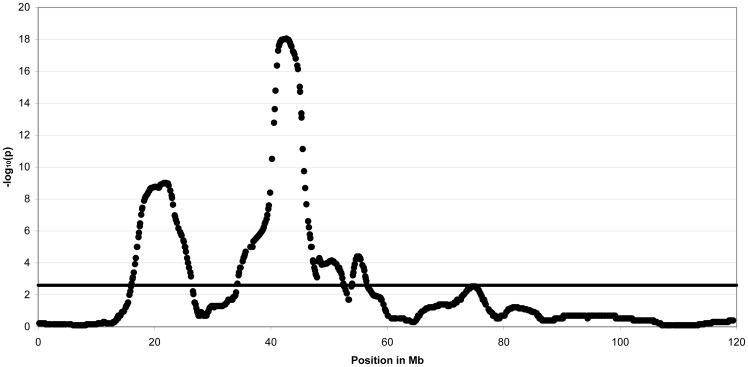
Non-parametric multipoint linkage analysis for guttural pouch tympany in German warmblood horses. Plot of −log_10_ chromosome-wide significant P-values (−log_10_P) for horse chromosome (ECA) 3 using non-parametric multipoint linkage analysis in five German warmblood families. P-values are genome-wide significant above the −log_10_P threshold value of 2.6. The maximum of the curve is at 41–43 Mb and represented by 14 SNPs.

**Figure 4:**
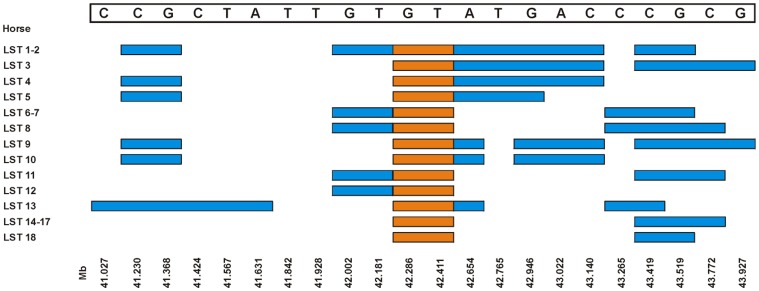
Homozygosity mapping in the linkage region (41–43 Mb) for guttural pouch tympany (GPT) in German warmblood horses on ECA3. Genotypes of the GPT-affected horses of warmblood families 1 and 2 were used to delimit the common homozygous block of SNPs in GPT-affected horses. This block of homozygous SNPs at 42.29–42.41 Mb is highlighted in orange.

For the German warmblood horses using 14,378 SNPs with an average spacing of 125 kb, the highest peak was located on ECA3 (Figure 3) and in addition to that, on ECA1, 18 and 30 regions with genome-wide significant linkage were found (Figure S2). The chromosomal region with the highest genome-wide significant linkage was located on ECA3, at 16–26 Mb and 34–55 Mb (Table S2). The highest peak was at 41–43 Mb indicating this region on ECA3 with the most common haplotype for affected foals. Homozygosity was observed in 98.5% of all affected German warmblood foals for a short haplotype block extending 125 kb at 42.3–42.4 Mb (Figure 4). However, 83.3% of all unaffected German warmblood horses were also homozygous. On ECA1, there were three linked regions at 21–24, 38–41 and at 79–87 Mb, on ECA18 at 72–78 Mb and on ECA30 at 26–27 Mb.

### Genome-wide Association Mapping

**Figure 5:**
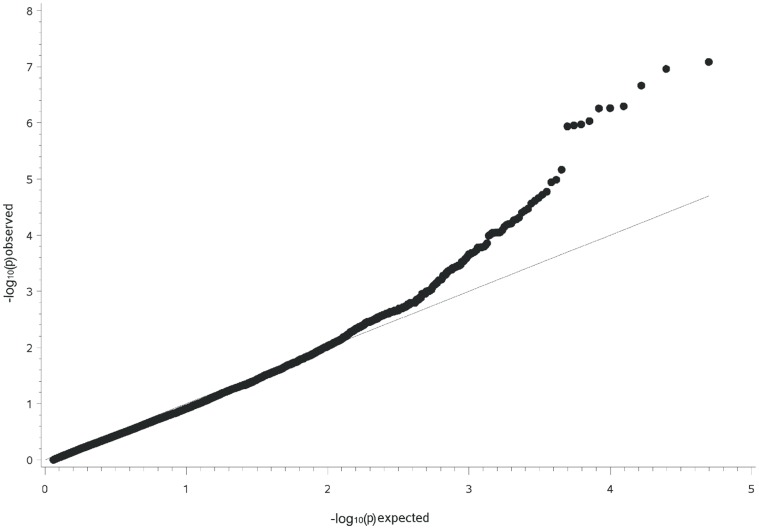
Q-Q plot of observed versus expected −log_10_P-values from a genome- wide association analysis for GPT in the Arabian. The quantile-quantile plot shows the expected distribution (solid line) and the observed −log_10_P-values plotted against the expected −log_10_P-values (black dots). The peak value (BIEC2-314665) is located at 64.17 Mb on horse chromosome 15 for the Arabian.

The genome-wide association study (GWAS) revealed for the Arabian a significant hit at a −log_10_P-value of 6.26 on ECA15 for the SNP BIEC2-314665 at 64.17 Mb (Figure S3). The P-value of the MLM analysis corrected for multiple testing using the Benjamini-Hochberg procedure yielded a P-value<0.05. The observed −log_10_P-values were plotted against the expected −log_10_P-values and the quantile-quantile plots indicated that the population stratification was eliminated through the identity-by-state (IBS) kinship matrix and the fixed effects of sex and inbreeding as far as possible (Figure 5). The SNP with the highest association was located in the intergenic region between *TTC27* and *BIRC6* at 64 Mb. The minor allele frequency (MAF) of the highly associated SNP BIEC2-314665 was 0.18 (G) for the GPT-affected Arabian and 0.65 (G) for controls. The genotype distribution in GPT-affected Arabian was 0.64 for A/A, 0.36 for A/G and 0.00 for G/G. In controls, the genotype distribution was 0.20 for A/A, 0.30 for A/G and 0.50 for G/G (Table 1). The odds ratio (OR) of the minor allele was 0.12 with 95% confidence intervals at 0.06–0.25. Pairwise linkage disequilibria (LD) among alleles for all loci in the significantly linked and associated regions were estimated using Haploview, version 4.2. A LD block with an aggregation of SNPs flanking the associated SNP BIEC2-314665 at 64 Mb with r^2^ = 0.3 could be detected on ECA15. The LD block was covering 86.9 kb. Further 15 LD blocks were at 62–63 Mb, 65 Mb and 66–67 Mb (Figure S4).

**Table 1:** Association of genotypes with guttural pouch tympany (GPT) in the Arabian.

Horses	MAF		Genotypic distribution		
			A/A	A/G	G/G
GPT-affected		0.18	0.64	0.36	0.00
Controls		0.65	0.20	0.30	0.50
All Arabian		0.48	0.36	0.33	0.31

Minor allele frequency (MAF) and distribution of the SNP alleles of BIEC2-314665 on ECA15 for the Arabian in the region of the highest association. The odds ratio of this SNP-allele is 0.12.

**Figure 6:**
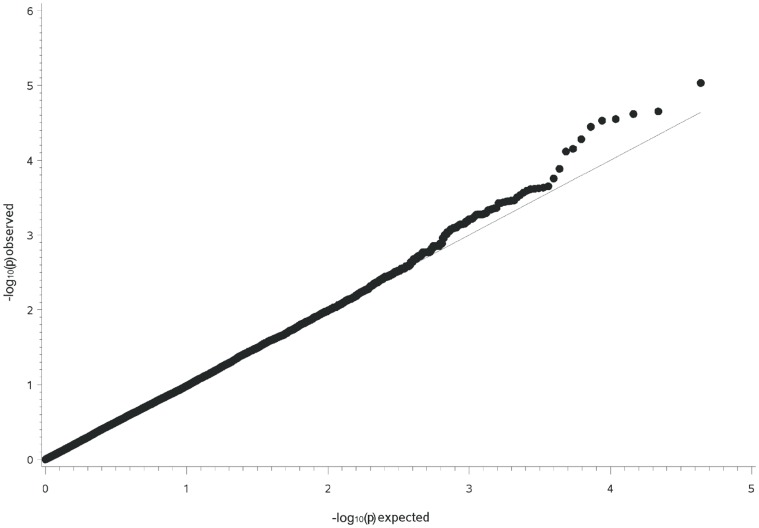
Q-Q plot of observed versus expected −log_10_P-values from a genome- wide association analysis for GPT in German warmblood horses. The quantile-quantile plot shows the expected distribution (solid line) and the observed −log_10_P-values plotted against the expected −log_10_P-values (black dots). The peak value (BIEC2-780830) is located at 51.86 Mb on horse chromosome 3 for the warmblood horses.

The GWAS for German warmblood showed unlike the highest peak of linkage an association at 51.86 Mb (−log_10_P = 5.4, P-value<0.05 after correcting for multiple testing using the Benjamini-Hochberg procedure) on ECA3, located within intron 1 of *ARHGAP24* (Figure S5). The quantile-quantile plots indicated that the population stratification was almost removed employing a mixed animal model (Figure 6). The SNP with the highest association on ECA3 (BIEC2-780830) had a small MAF of 0.05 and thus, a few GPT-affected horses had a large influence on the genotypic distribution among affected and control animals. No further significantly associated SNPs could be detected within the linkage regions on ECA1, 18 and 30 or on other horse chromosomes. The genotypic distribution of the associated SNP BIEC2-780830 at 52 Mb was 0.0 (T) for GPT-affected warmblood horses and 0.07 (T) for controls (Table 2). All GPT-affected animals carried the genotype C/C and in controls, genotype frequencies were 0.15 (C/T) and 0.85 (C/C). On ECA3, there were 23 LD blocks at 45 Mb, in the region of 47–48 Mb and at 50 Mb (Figure S6). There were no SNPs in strong LD in the vicinity of the associated SNP at 52 Mb. BIEC2-780830 resides in a LD block of 82.4 kb with an r^2^ = 0.03 and LOD = 2.42 (D’ = 0.5). The LD of BIEC2-780830 to the neighbouring SNPs was r^2^ = 0.02 and 0.04, respectively.

**Table 2:** Association of genotypes with guttural pouch tympany (GPT) in German warmblood horses.

Horses	MAF	Genotypic distribution	
		C/T	C/C
GPT-affected	0.00	0.00	1.00
Controls	0.07	0.15	0.85
All warmblood horses	0.05	0.10	0.90

Minor allele frequency (MAF) and distribution of the SNP alleles of BIEC2-780830 on ECA3 for warmblood horses in the region of the highest association.

## Discussion

The aim of this work was to identify linked and associated regions for a major gene causing GPT. The previously reported QTL on ECA15 was confirmed for the Arabian horse through genome-wide linkage and association analyses using the Illumina equine SNP50 beadchip. In the present linkage analysis, the position of the QTL was narrowed down to approximately 2 Mb at the proximal end of the QTL in the previous analysis. The higher marker density and the high information content of haplotypes through multipoint linkage analyses allowed us to adjust the location of the responsible region in Arabian horses using genome-wide significant P-values. The association of the SNP BIEC2-314665 with GPT confirmed this region. Therefore, we assume that the region at 64–65 Mb on ECA15 contains the major gene for the Arabian horse. For the German warmblood horses, the very high linkage peak at 41–43 Mb on ECA3 and a nearly homozygous 125 kb block at 42 Mb in affected foals suggest that the major gene for GPT may reside in this segment or nearby to this segment. For the linkage and association analyses, the number of GPT-affected foals was similar in both breeds analysed here. Therefore, we assume that differences in the power of the analyses to detect significant linkage and association were small among these two breeds. Despite the larger number of affected foals and controls in the German warmblood, we could not find significant linkage or association for GPT on ECA15 for the warmblood horses. For the Arabian horse, we can not preclude that a further locus influencing GPT may be located on ECA3 even if GWAS did not support this assumption and linkage analysis did not show genome-wide significance.

Summarizing these results, we identified two different breed-specific QTL for GPT. Possibly, these QTL could harbour genes linked through their pathways or regulatory effects on other genes.

A positional candidate gene on ECA15 is *TTC27*, the *tetratricopeptide repeat domain 27* (63.99–64.16 Mb). The tetratricopeptide repeat (TPR) motif is a protein-protein interaction module found in multiple copies in a number of functionally different proteins that facilitates specific interactions with a partner protein(s). Most TPR-containing proteins are associated with multiprotein complexes, and there is extensive evidence indicating that TPR motifs are important to the functioning of chaperone, cell-cycle, transcription, and protein transport complexes [8]. It is possible that an important trigger for the GPT is influenced that way. Another cell specific positional candidate gene is *BIRC6* (64.18–64.40 Mb). It is essential for cell viability and inhibits apoptosis by facilitating the degradation of apoptotic proteins.

For the German warmblood horses, a very high linkage signal was evident at 41–43 Mb on ECA3. The linkage peak region did not exactly fit with the associated SNP but was displaced proximally. A likely reason for this result could be that the associated region is smaller than in the Arabian horse due to the different warmblood breeds included and therefore, the SNP density is too low for identification of a highly associated region. The data set of German warmblood horses contained horses of different breeds like Hanoverian, Oldenburg, Trakehner, Holstein and others. A low MAF of the associated SNP and a low LD with adjacent SNPs may indicate a small GPT-associated region which is not well covered by informative SNPs. A further reason for missing coincidence of the highly linked region with the associated SNP could be that only very few animals possess a larger LD block harbouring the GPT-causing gene and in the other animals recombinations in preceding generations and intermixing with different warmblood breeds lead to a very small LD block with the GPT-causing gene. In this situation, the LD block is hardly detected because common SNPs are not associated with GPT and only rare SNP alleles may show significant associations. However, we can not preclude that a small LD block in association with GPT may be detected at another location within the linked region when the SNP density is increased. Within the highly linked region, it is hard to detect a clear candidate gene as most of the genes annotated in EquCab2.0 are novel non-coding pseudogenes or a retrotransposed pseudogene or a novel protein coding gene.

Assuming that a failure of the mechanism of the flap at the orifice and the *tuba auditiva* is the reason for the tympany, there are different ways of interfering by various genes. A putative candidate gene on ECA3 influencing cartilage formation is *IBSP*, the *integrin-binding sialoprotein* (50.04–50.05 Mb). The cartilage is an important reinforcing structure for the *tuba auditiva* and at the medial side for the flap at the orifice. Eustachian tube dysfunction also plays an important role in the otitis media in men [9]. The candidate genes *SLC39A8*, *solute carrier family 39* (zinc transporter), *member 8* (37.58–37.62) on ECA3 and *SLC30A6*, *solute carrier family 30* (zinc transporter), *member 6* (64.54–64.58) on ECA15 could possibly be involved. Both genes belong to a subfamily of proteins (GO:0071577) that show structural characteristics of zinc transporters. Zinc is an essential cofactor for hundreds of enzymes. It is involved in protein, nucleic acid, carbohydrate, and lipid metabolism, as well as in the control of gene transcription, growth, development, and differentiation [10].

## Materials and Methods

### Ethics Statement

All animal work has been conducted according to the national and international guidelines for animal welfare. The EDTA-blood sampling at the University of Veterinary Medicine Hannover was approved by the Lower Saxony state veterinary office Niedersächsisches Landesamt für Verbraucherschutz und Lebensmittelsicherheit, Oldenburg, Germany (registration number 509c-42502-01A60).

### Animals

Clinical data and EDTA-blood samples of GPT-affected foals were collected in the Clinic for Horses of the University of Veterinary Medicine Hannover. All cases of GPT were clinically and endoscopically examined and surgically treated. We could not observe substantial differences in the type of signs for GPT between fillies and colts as well as between the two breeds. A total of 458 samples including 70 GPT-affected and 388 unaffected horses were available for the genome-wide association study. The data included 19 GPT-affected Arabian and 29 GPT-affected German warmblood fillies as well as 14 GPT-affected Arabian and eight GPT-affected German warmblood colts (Table S3). Unaffected horses comprised 52 Arabian and 336 German warmblood horses. We collected the pedigree data for the GPT-affected horses to identify their family structure (Figure S7). Linkage analyses were performed using five Arabian and five German warmblood families with 56 Arabian and 42 German warmblood samples (Table S4) including 32 GPT-affected Arabian and 36 GPT-affected German warmblood horses. All horses with genotypes used for the linkage study were also contained in the GWAS.

### Genotyping

For genotyping we isolated genomic DNA from EDTA blood samples or hair roots using standard methods with RBC (Red Blood Cell) lysis buffer and SE (sodium EDTA) buffer. The DNA concentration of the samples was adjusted to 50 ng/µl. DNA concentration was determined using the Nanodrop ND-1000 (Peqlab Biotechnology, Erlangen, Germany).

Genotyping was performed using the Illumina equine SNP50 beadchip including 54,602 SNPs (single nucleotide polymorphisms) using standard procedures as recommended by the manufacturer. Data were analyzed using the genotyping module version 3.2.32 of the BeadStudio program (Illumina). With the help of BeadStudio software a cluster file was generated.

### Data Analysis

A total of 49,797 SNPs with a minor allele frequency (MAF) of >0.05, a call rate of >90% and an average MAF of 0.25 were left for the analysis of the Arabian. For German warmblood horses, 43,440 SNPs were available under the same restrictions. Data quality control was done using PLINK version 1.07 (http://pngu.mgh.harvard.edu/purcell/plink/Purcell et al. 2007) and SAS/Genetics version 9.3 (Statistical Analysis System, Cary, NC, USA, 2011). The genotyping rate per animal was set >0.98. The threshold for Hardy-Weinberg equilibrium was set to P = 10^−7^.

Whole genome scans were performed using multipoint non-parametric linkage analyses for each of both breeds separately. Linkage analyses were done using the software MERLIN software (multipoint engine for rapid likelihood inference, version 1.1.2). Linkage between GPT and SNPs was estimated through the proportion of alleles identical-by-descent (IBD) for affected horses. The NPL (non parametric linkage) pairs-statistics by Whittemore and Halpern [11] and the Z-means and LOD-scores according to Kong and Cox [12] were used to identify the alleles and haplotypes shared by affected family members. We employed two marker sets with different spacing of SNPs. The first marker set included 4473 SNPs with an average distance of 500 kb and the second marker set 14,378 SNPs with an average spacing of 125 kb. Genome-wide probabilities were obtained by applying a Bonferroni correction: P_genome-wide_  =  (1–P_chromosome-wide_)^1/r^, where r is the length of the respective horse chromosome in Mb (185.8 Mb for ECA1, 119.5 Mb for ECA3, 91.6 Mb for ECA15, 82.5 Mb for ECA18, 30.1 Mb for ECA30) divided by the total equine genome length (2,680 Mb).

For the genome-wide association study, a case-control association analysis was performed using 49,797 SNPs for Arabian and 43,440 SNPs for German warmblood horses. A mixed linear model (MLM) was employed for the genome-wide association analysis in order to control for a complex population structure, sex and inbreeding of the horses. MLM analyses were performed using TASSEL (Trait Analysis by Association, Evolution and Linkage), version 2.1, a software appropriate for association mapping of complex traits in diverse samples. The Q-matrix to explain population structure, the identity-by-state (IBS) kinship matrix and the degree of inbreeding (deficit of heterozygotes, F_IS_) were estimated using equidistantly distributed SNPs at a pairwise linkage disequilibrium (r^2^) <0.2. This pruned set of SNPs was generated jointly for both breeds using a window size of 100 SNPs, a shift by five SNPs at each step and an r^2^ <0.2. The number of SNPs contained of this subset was 4,473. The Q-matrix was determined using STRUCTURE, version 2.3.3 [13]. For a value of k = 5, the likelihood of the data could be maximized for each of both breeds. The MLM and STRUCTURE analyses were done for each breed separately. As the cryptic data structure revealed via STRUCTURE did not influence the outcome of the GWAS, we omitted these effects in the final model. The final model included the effects of the degree of inbreeding as covariate, sex as class variable, the random animal effects parameterized via the IBS kinship matrix and the respective genotype effect. Quantile-quantile (Q-Q) plots for observed versus expected –log_10_P-values were constructed to control for population stratification and to visualize significant SNP genotype effects. P-values from MLM analyses were corrected using the Benjamini-Hochberg procedure [14]. For the genomic regions containing SNPs with the highest −log_10_P-values, we estimated the size of haplotypes for GPT-affected horses using LD and haplotype block analysis by Haploview, version 4.2 [15].

## Supporting Information

Figure S1 **Manhattan-plot of the −log_10_P-values from the multipoint non-parametric linkage analysis for the Arabian.** The highest peak is located at 47–81 Mb on ECA15 and the next highest peak on ECA3. Genome-wide significant linkage was only found for ECA15.
(DOC)

Figure S2 **Manhattan-plot of the −log_10_P-values from the multipoint non-parametric linkage analysis for the German warmblood horses.** The highest peak is located at 34–55 Mb on ECA3 and the next highest peaks on ECA1, 18 and 30. Genome-wide significant linkage could be detected in all these peak regions.
(DOC)

Figure S3 **P-values from the genome-wide association analysis for the Arabian.** Distribution of –log_10_P-values in the region of 60–67 Mb on ECA15. The lower panel shows the genes depicted by black boxes below the x-axis located in the region of interest. The SNP with the strongest association (BIEC2-314665) is located in between *TTC27* and *BIRC6*.
(DOC)

Figure S4 **Linkage disequilibria (LD) for the SNP alleles at 60–67 Mb on ECA15 for Arabian.** The LD display presents Hedrige’s multiallelic D, which represent the degree of LD between two blocks. Red fields display LOD≥2 (D’ = 1), shades of red show the same LOD with D’<1. White and blue fields display LOD<2 with D’<1 and D’ = 1, respectively. The highly associated SNP BIEC2-314665 at 64 Mb with r^2^ = 0.3 is flanked by an aggregation of SNPs forming an LD block. Further 15 LD blocks can be detected at 62–63 Mb, 65 Mb and 66–67 Mb.
(DOC)

Figure S5 **P-values from the genome-wide association analysis for German warmblood horses.** Distribution of –log_10_P-values in the region of 40–53 Mb on ECA3. The lower panel shows the genes depicted by black boxes below the x-axis located in the region of interest. The SNP showing the strongest association (BIEC2-780830) is highlighted. BIEC2-780830 is located within intron 1 of *ARHGAP24*.
(DOC)

Figure S6 **Linkage disequilibria (LD) for the SNP alleles at 45–52 Mb on ECA3 for German warmblood horses.** The LD display presents Hedrige’s multiallelic D, which represent the degree of LD between two blocks. Red fields display LOD≥2 (D’ = 1), shades of red show the same LOD with D’<1. White and blue fields display LOD<2 with D’<1 and D’ = 1, respectively. The highly associated SNP BIEC2-780830 at 52 Mb shows an LD block of 82.4 Kb with r^2^ = 0.029 and LOD = 2.42 (D’ = 0.5).
(DOC)

Figure S7 **Pedigrees of the five Arabian and five German warmblood families used in multipoint linkage analyses.**
(DOC)

Table S1 **Results from the genome-wide linkage analysis (ECA15).** Multipoint chromosome-wide significant Zmeans and LOD scores, their chromosome-wide P-values (P_z_, P_L_) and positions in Mb for all Arabian horses. Genome-wide significant P-values<0.05 at 64–65 are in bold.
(DOC)

Table S2 **Results from the genome-wide linkage analysis (ECA3).** Multipoint chromosome-wide significant Zmeans and LOD scores, their chromosome-wide P-values (P_z_, P_L_) and positions in Mb for all German warmblood horses. Genome-wide significant P-values<0.05 at 34–55 Mb are in bold.
(DOC)

Table S3 **Survey on all animals genotyped for the genome-wide association study and by foals affected by guttural pouch tympany and unaffected animals in total and by breed and sex.**
(DOC)

Table S4 **Arabian and German warmblood horses used for the multipoint linkage analysis.** Number of animals used to construct pedigrees and number of genotyped samples using the Illumina equine SNP50 beadchip by families and their affection status, number of affected foals and their distribution by sex and family and in total.
(DOC)
