# Supplementary material for: Genome-Wide Linkage and Association Analysis Identifies Major Gene Loci for Guttural Pouch Tympany in Arabian and German Warmblood Horses
Source: PLoS One. 2012 Jul 27;7(7):e41640. doi: 10.1371/journal.pone.0041640 (PMC3407181; doi:10.1371/journal.pone.0041640)
Supplement: Figure S1 — Manhattan-plot of the −log10P-values from the multipoint non-parametric linkage analysis for the Arabian. The highest peak is located at 47–81 Mb on ECA15 and the next highest peak on ECA3. Genome-wide significant linkage was only found for ECA15. (DOC) [file pone.0041640.s001.doc]

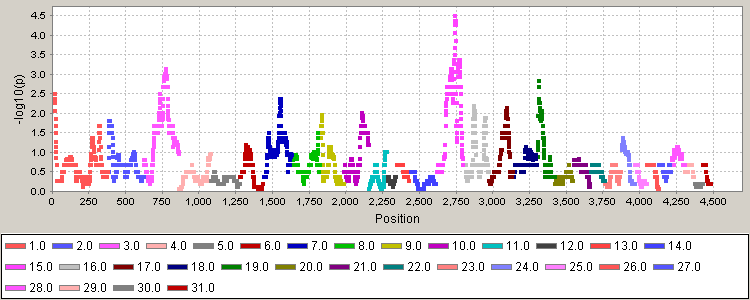


**Figure S1. Manhattan-plot of the -log_10_P-values from the multipoint non-parametric linkage analysis for the Arabian.** The highest peak is located at 47-81 Mb on ECA15 and the next highest peak on ECA3. Genome-wide significant linkage was only found for ECA15.
