# Supplementary material for: Genome-Wide Linkage and Association Analysis Identifies Major Gene Loci for Guttural Pouch Tympany in Arabian and German Warmblood Horses
Source: PLoS One. 2012 Jul 27;7(7):e41640. doi: 10.1371/journal.pone.0041640 (PMC3407181; doi:10.1371/journal.pone.0041640)
Supplement: Figure S2 — Manhattan-plot of the −log10P-values from the multipoint non-parametric linkage analysis for the German warmblood horses. The highest peak is located at 34–55 Mb on ECA3 and the next highest peaks on ECA1, 18 and 30. Genome-wide significant linkage could be detected in all these peak regions. (DOC) [file pone.0041640.s002.doc]

**
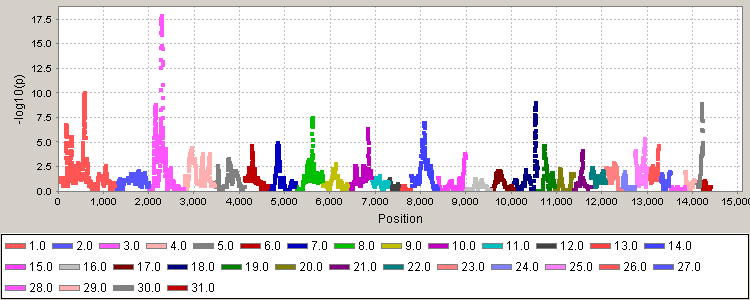
**

**Figure S2. Manhattan-plot of the -log_10_P-values from the multipoint non-parametric linkage analysis for the German warmblood horses.** The highest peak is located at 34-55 Mb on ECA3 and the next highest peaks on ECA1, 18 and 30. Genome-wide significant linkage could be detected in all these peak regions.
