# Supplementary material for: Genome-Wide Linkage and Association Analysis Identifies Major Gene Loci for Guttural Pouch Tympany in Arabian and German Warmblood Horses
Source: PLoS One. 2012 Jul 27;7(7):e41640. doi: 10.1371/journal.pone.0041640 (PMC3407181; doi:10.1371/journal.pone.0041640)
Supplement: Figure S3 — P-values from the genome-wide association analysis for the Arabian. Distribution of –log10P-values in the region of 60–67 Mb on ECA15. The lower panel shows the genes depicted by black boxes below the x-axis located in the region of interest. The SNP with the strongest association (BIEC2-314665) is located in between TTC27 and BIRC6. (DOC) [file pone.0041640.s003.doc]

**
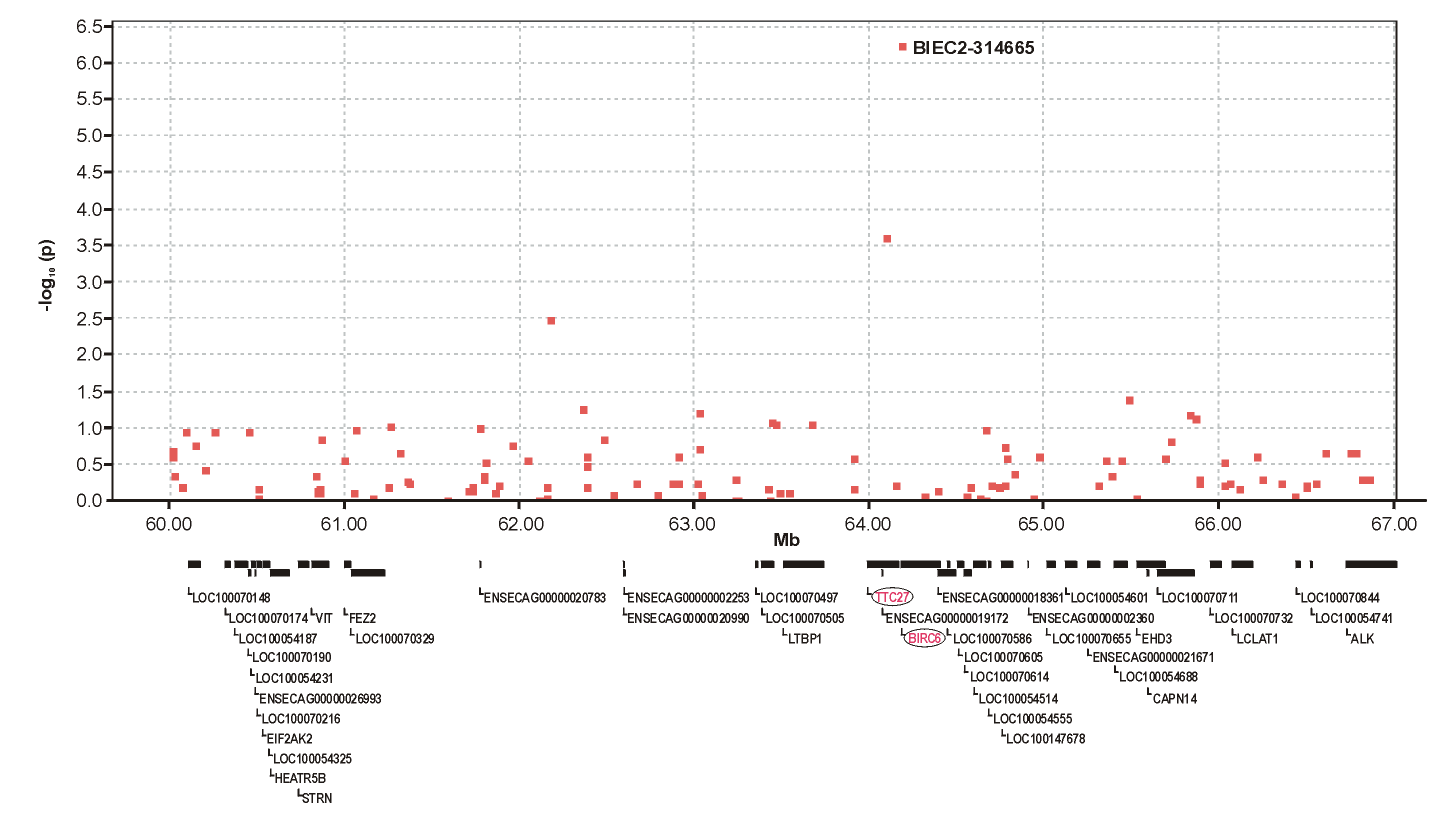
**

**Figure S3. P-values from the genome-wide association analysis for the Arabian.** Distribution of –log_10_P-values in the region of 60-67 Mb on ECA15. The lower panel shows the genes depicted by black boxes below the x-axis located in the region of interest. The SNP with the strongest association (BIEC2-314665) is located in between *TTC27* and *BIRC6*.
