# Supplementary material for: Genome-Wide Linkage and Association Analysis Identifies Major Gene Loci for Guttural Pouch Tympany in Arabian and German Warmblood Horses
Source: PLoS One. 2012 Jul 27;7(7):e41640. doi: 10.1371/journal.pone.0041640 (PMC3407181; doi:10.1371/journal.pone.0041640)
Supplement: Figure S4 — Linkage disequilibria (LD) for the SNP alleles at 60–67 Mb on ECA15 for Arabian. The LD display presents Hedrige’s multiallelic D, which represent the degree of LD between two blocks. Red fields display LOD≥2 (D’ = 1), shades of red show the same LOD with D’<1. White and blue fields display LOD<2 with D’<1 and D’ = 1, respectively. The highly associated SNP BIEC2-314665 at 64 Mb with r2 = 0.3 is flanked by an aggregation of SNPs forming an LD block. Further 15 LD blocks can be detected at 62–63 Mb, 65 Mb and 66–67 Mb. (DOC) [file pone.0041640.s004.doc]

**
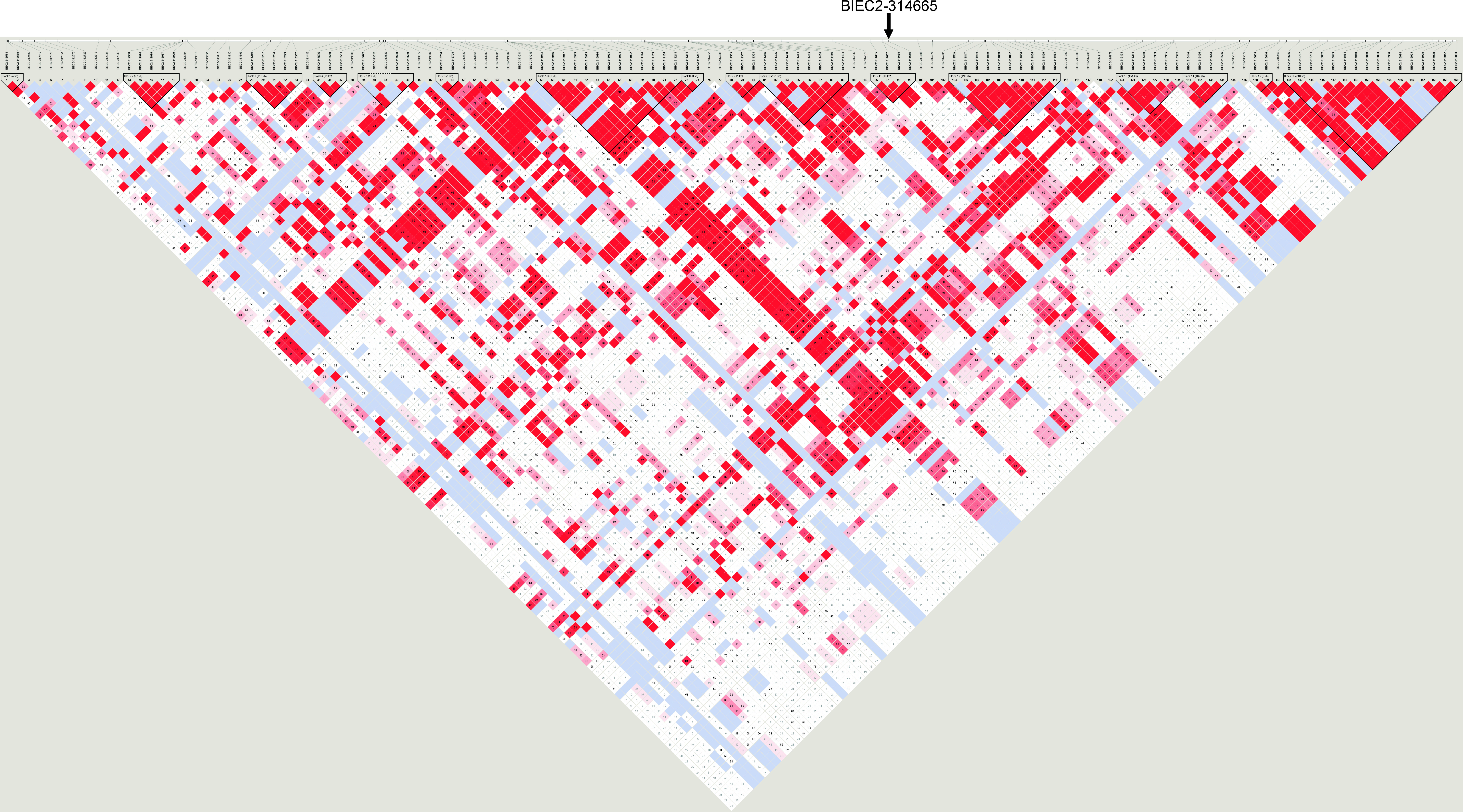
**

**Figure S4. Linkage disequilibria (LD) for the SNP alleles at 60-67 Mb on ECA15 for Arabian**. The LD display presents Hedrige’s multiallelic D, which represent the degree of LD between two blocks. Red fields display LOD≥2 (D’=1), shades of red show the same LOD with D’<1. White and blue fields display LOD<2 with D’<1 and D’=1, respectively. The highly associated SNP BIEC2-314665 at 64 Mb with r^2^=0.3 is flanked by an aggregation of SNPs forming an LD block. Further 15 LD blocks can be detected at 62-63 Mb, 65 Mb and 66-67 Mb.
