# Supplementary material for: Genome-Wide Linkage and Association Analysis Identifies Major Gene Loci for Guttural Pouch Tympany in Arabian and German Warmblood Horses
Source: PLoS One. 2012 Jul 27;7(7):e41640. doi: 10.1371/journal.pone.0041640 (PMC3407181; doi:10.1371/journal.pone.0041640)
Supplement: Figure S5 — P-values from the genome-wide association analysis for German warmblood horses. Distribution of –log10P-values in the region of 40–53 Mb on ECA3. The lower panel shows the genes depicted by black boxes below the x-axis located in the region of interest. The SNP showing the strongest association (BIEC2-780830) is highlighted. BIEC2-780830 is located within intron 1 of ARHGAP24. (DOC) [file pone.0041640.s005.doc]

**
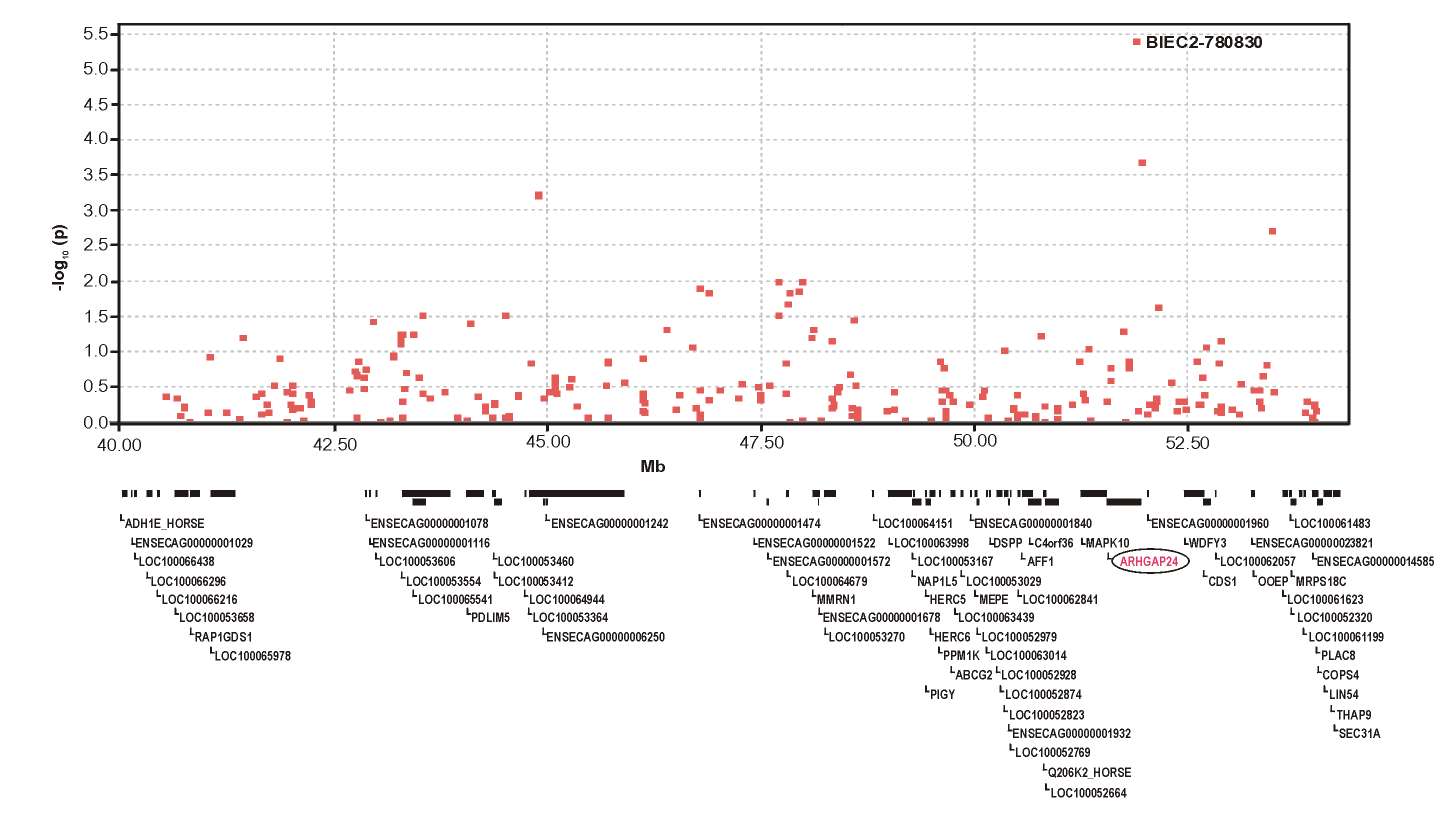
**

**Figure S5. P-values from the genome-wide association analysis for German warmblood horses.** Distribution of –log_10_P-values in the region of 40-53 Mb on ECA3. The lower panel shows the genes depicted by black boxes below the x-axis located in the region of interest. The SNP showing the strongest association (BIEC2-780830) is highlighted. BIEC2-780830 is located within intron 1 of *ARHGAP24*.
