# Supplementary material for: Genome-Wide Linkage and Association Analysis Identifies Major Gene Loci for Guttural Pouch Tympany in Arabian and German Warmblood Horses
Source: PLoS One. 2012 Jul 27;7(7):e41640. doi: 10.1371/journal.pone.0041640 (PMC3407181; doi:10.1371/journal.pone.0041640)
Supplement: Figure S6 — Linkage disequilibria (LD) for the SNP alleles at 45–52 Mb on ECA3 for German warmblood horses. The LD display presents Hedrige’s multiallelic D, which represent the degree of LD between two blocks. Red fields display LOD≥2 (D’ = 1), shades of red show the same LOD with D’<1. White and blue fields display LOD<2 with D’<1 and D’ = 1, respectively. The highly associated SNP BIEC2-780830 at 52 Mb shows an LD block of 82.4 Kb with r2 = 0.029 and LOD = 2.42 (D’ = 0.5). (DOC) [file pone.0041640.s006.doc]

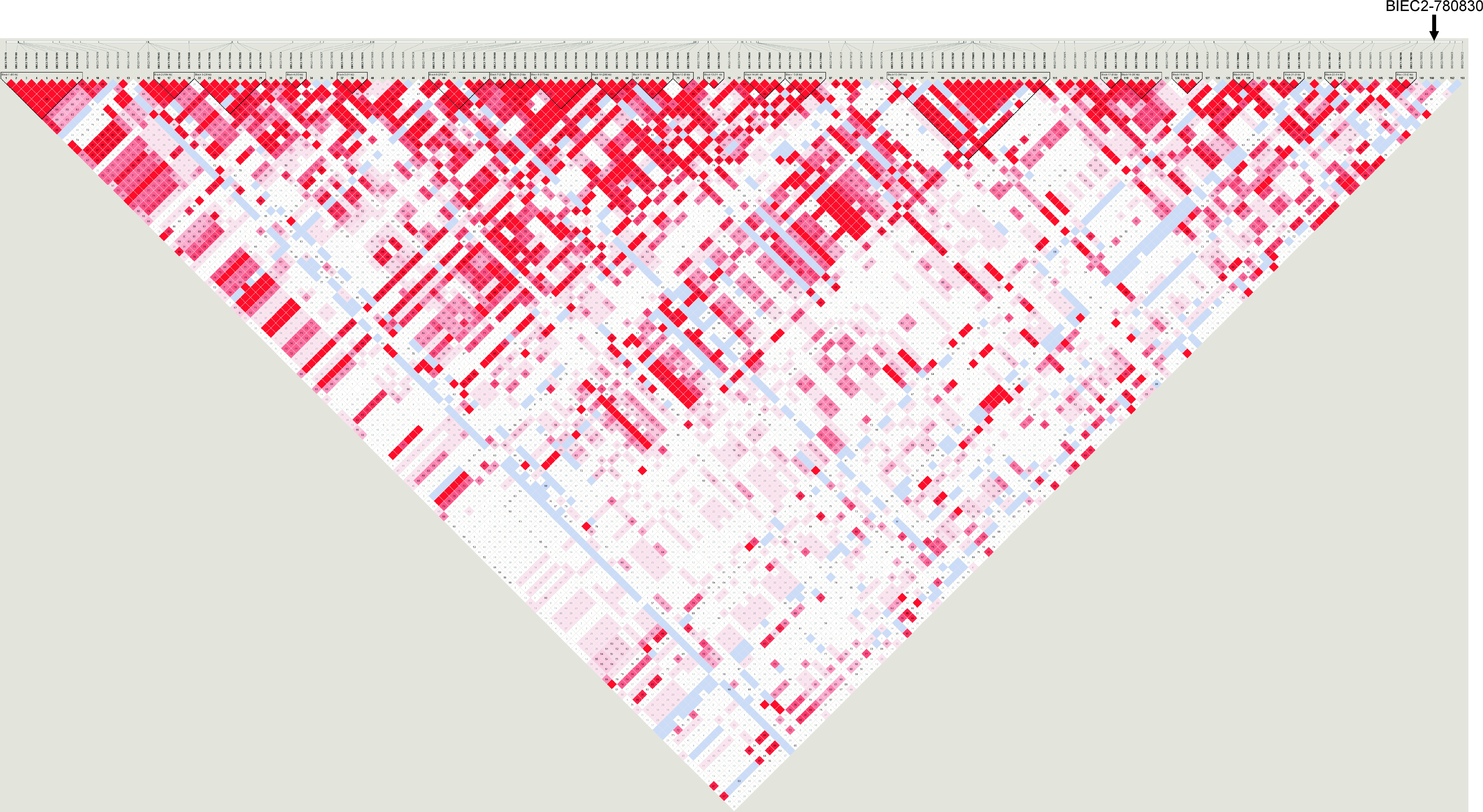


**Figure S6. Linkage disequilibria (LD) for the SNP alleles at 45-52 Mb on ECA3 for German warmblood horses.** The LD display presents Hedrige’s multiallelic D, which represent the degree of LD between two blocks. Red fields display LOD≥2 (D’=1), shades of red show the same LOD with D’<1. White and blue fields display LOD<2 with D’<1 and D’=1, respectively. The highly associated SNP BIEC2-780830 at 52 Mb shows an LD block of 82.4 Kb with r^2^ =0.029 and LOD=2.42 (D’=0.5).
