# Supplementary material for: Genome-Wide Linkage and Association Analysis Identifies Major Gene Loci for Guttural Pouch Tympany in Arabian and German Warmblood Horses
Source: PLoS One. 2012 Jul 27;7(7):e41640. doi: 10.1371/journal.pone.0041640 (PMC3407181; doi:10.1371/journal.pone.0041640)

**Figure S7. Pedigrees of the five Arabian and five German warmblood families used in multipoint linkage analyses.**

**
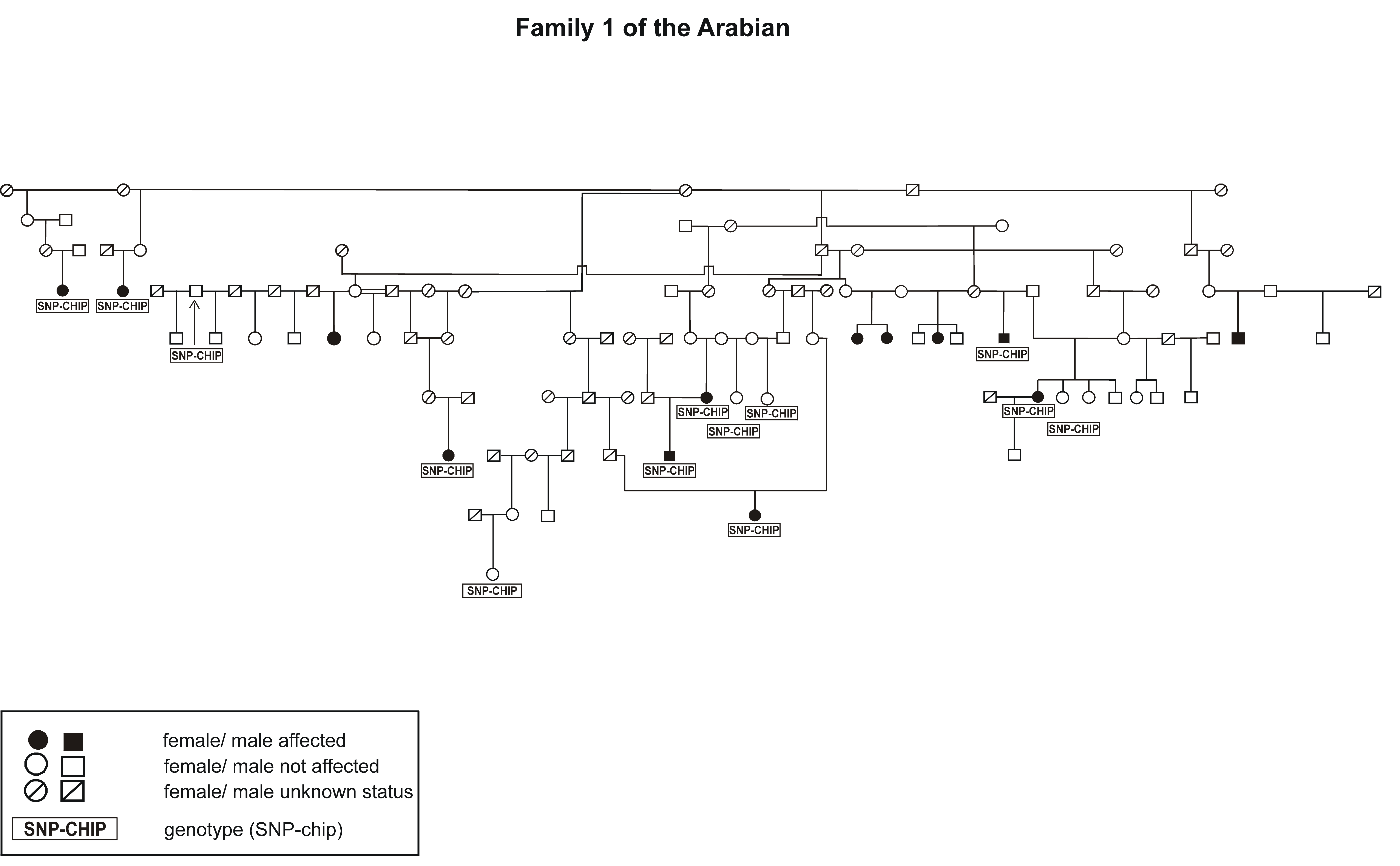
**


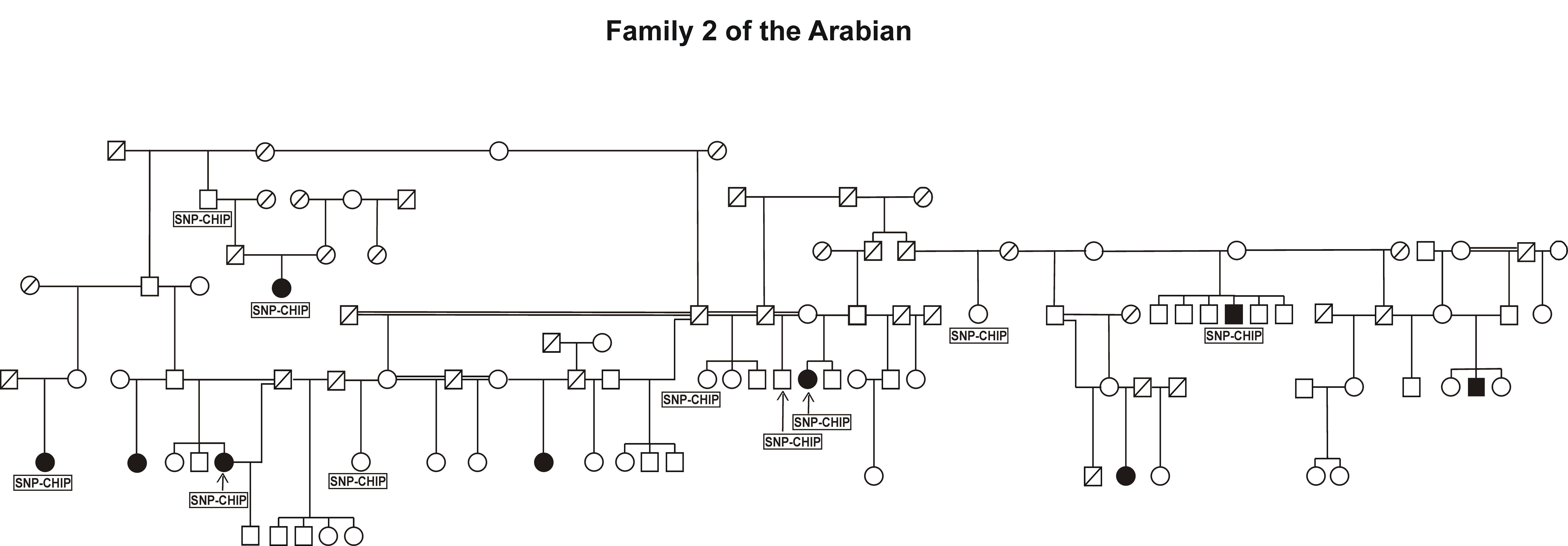


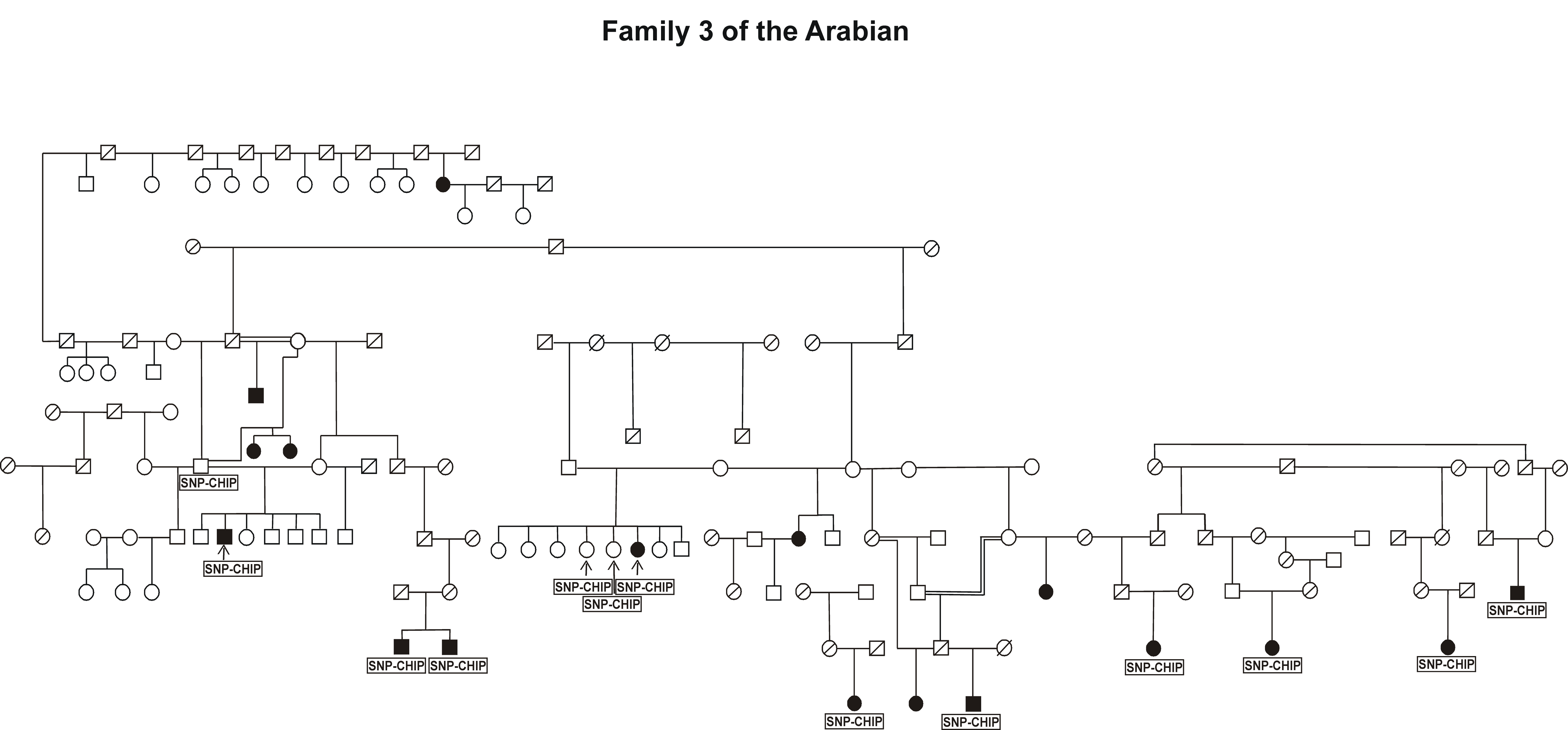


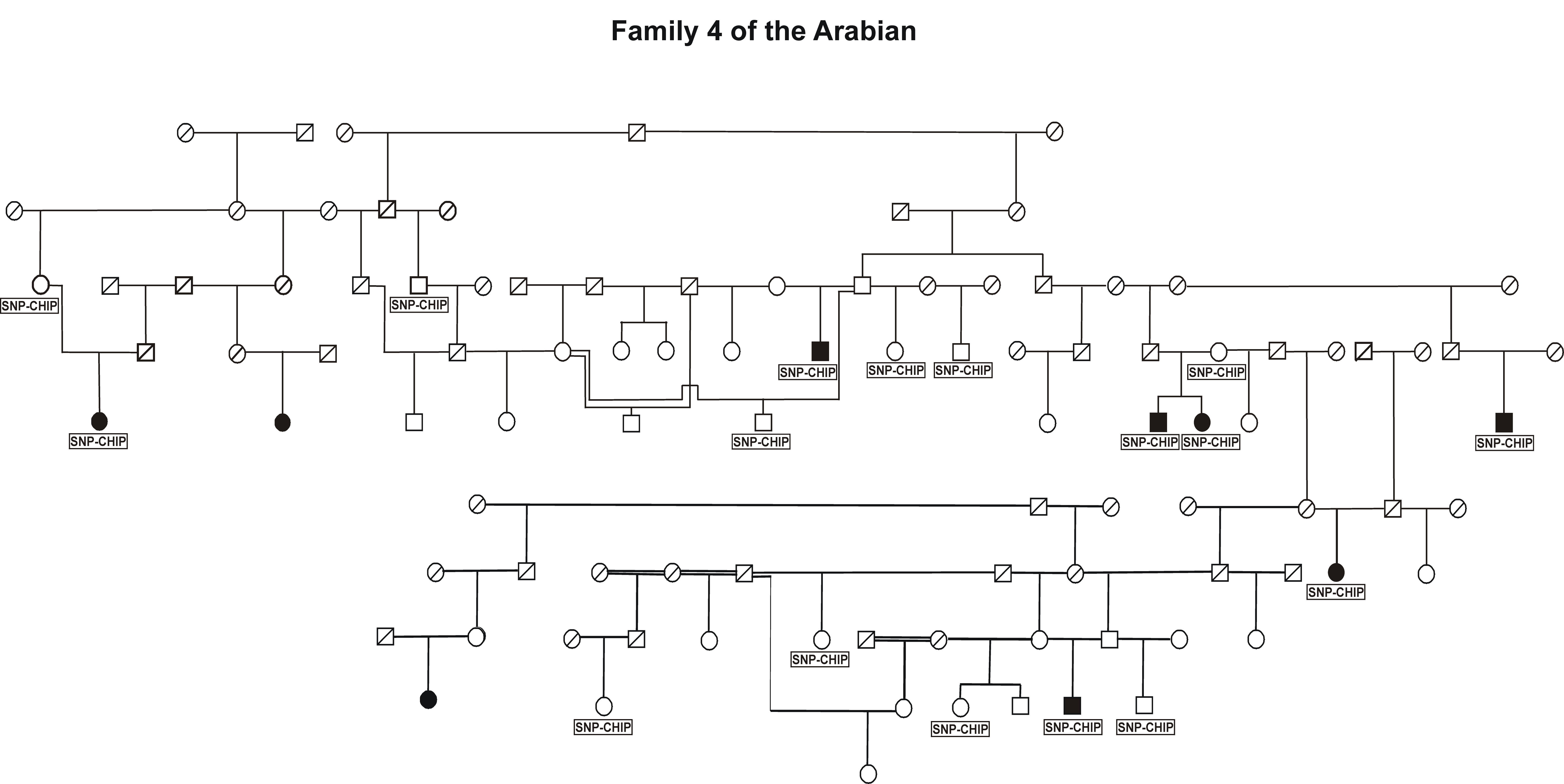


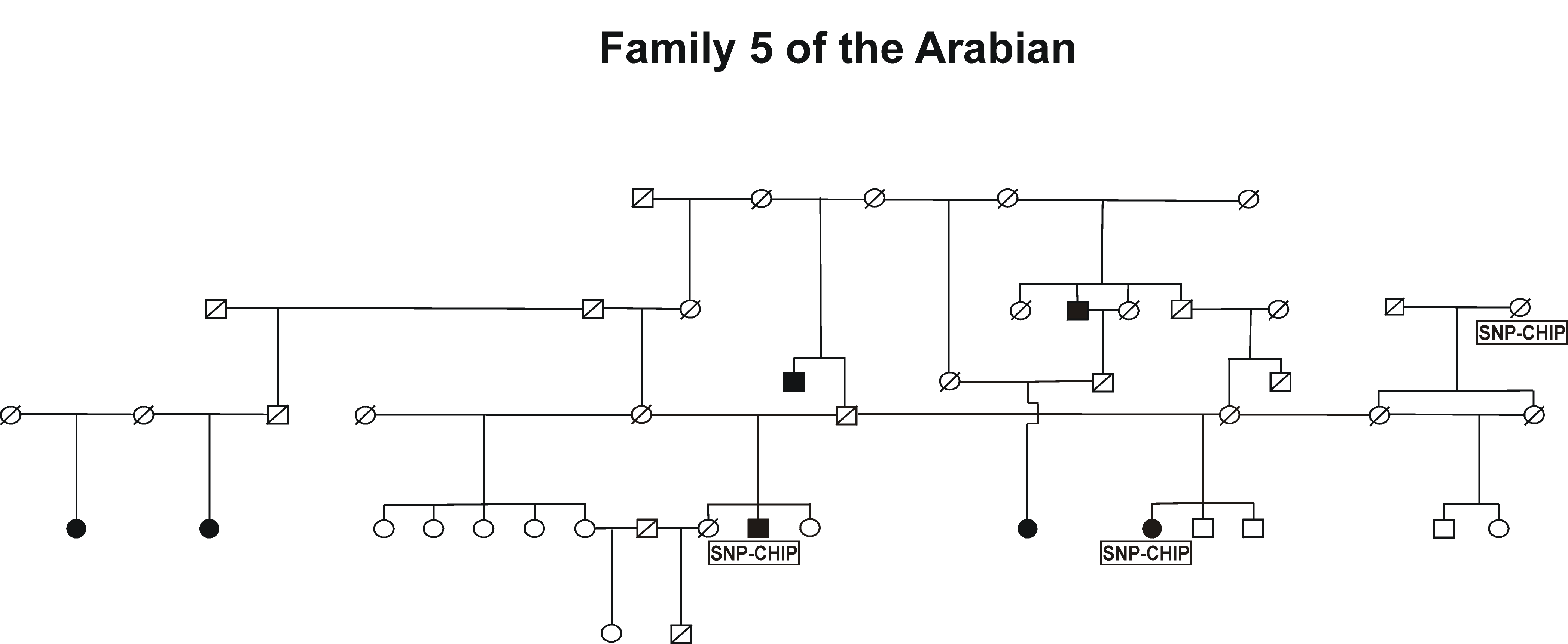


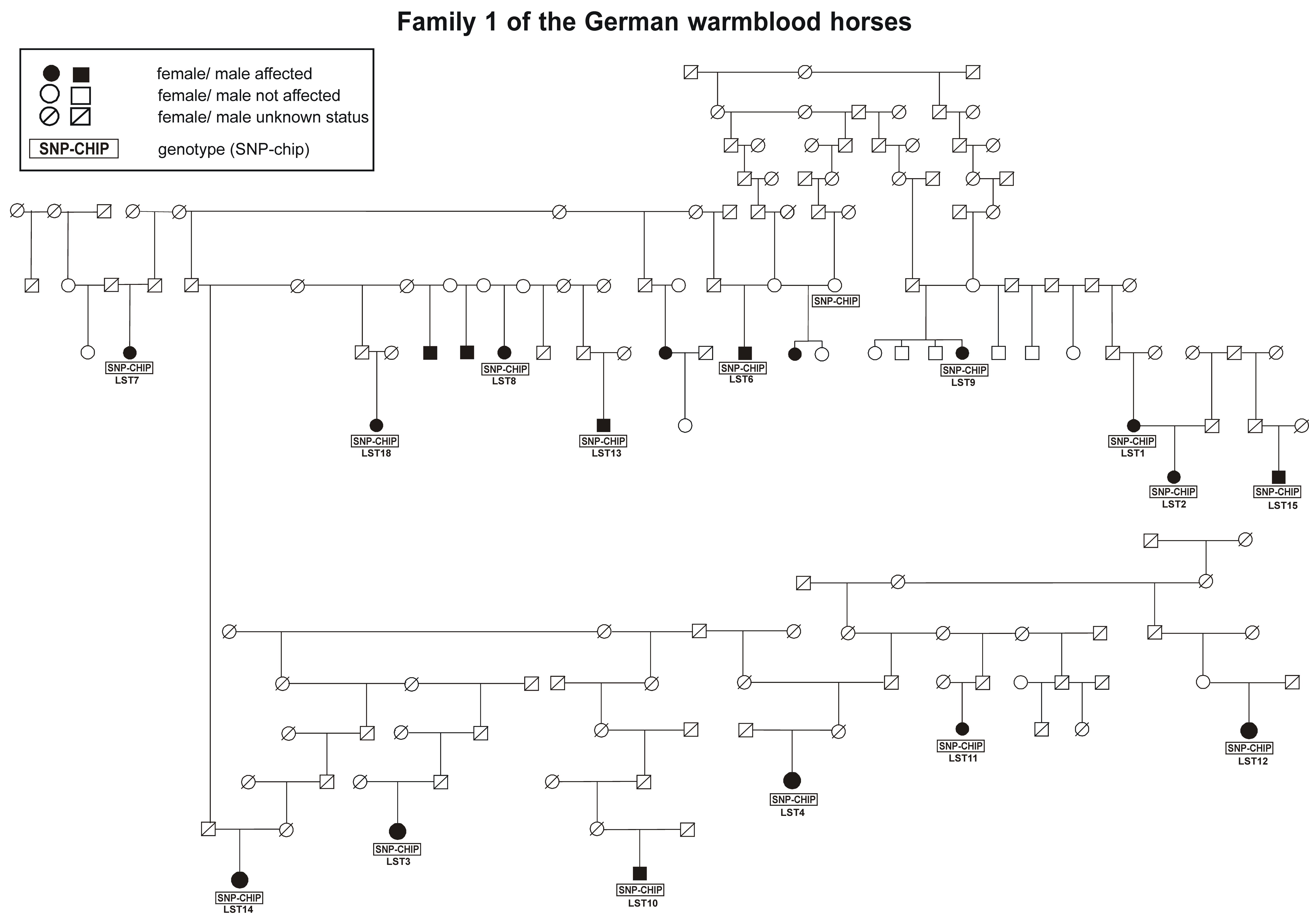


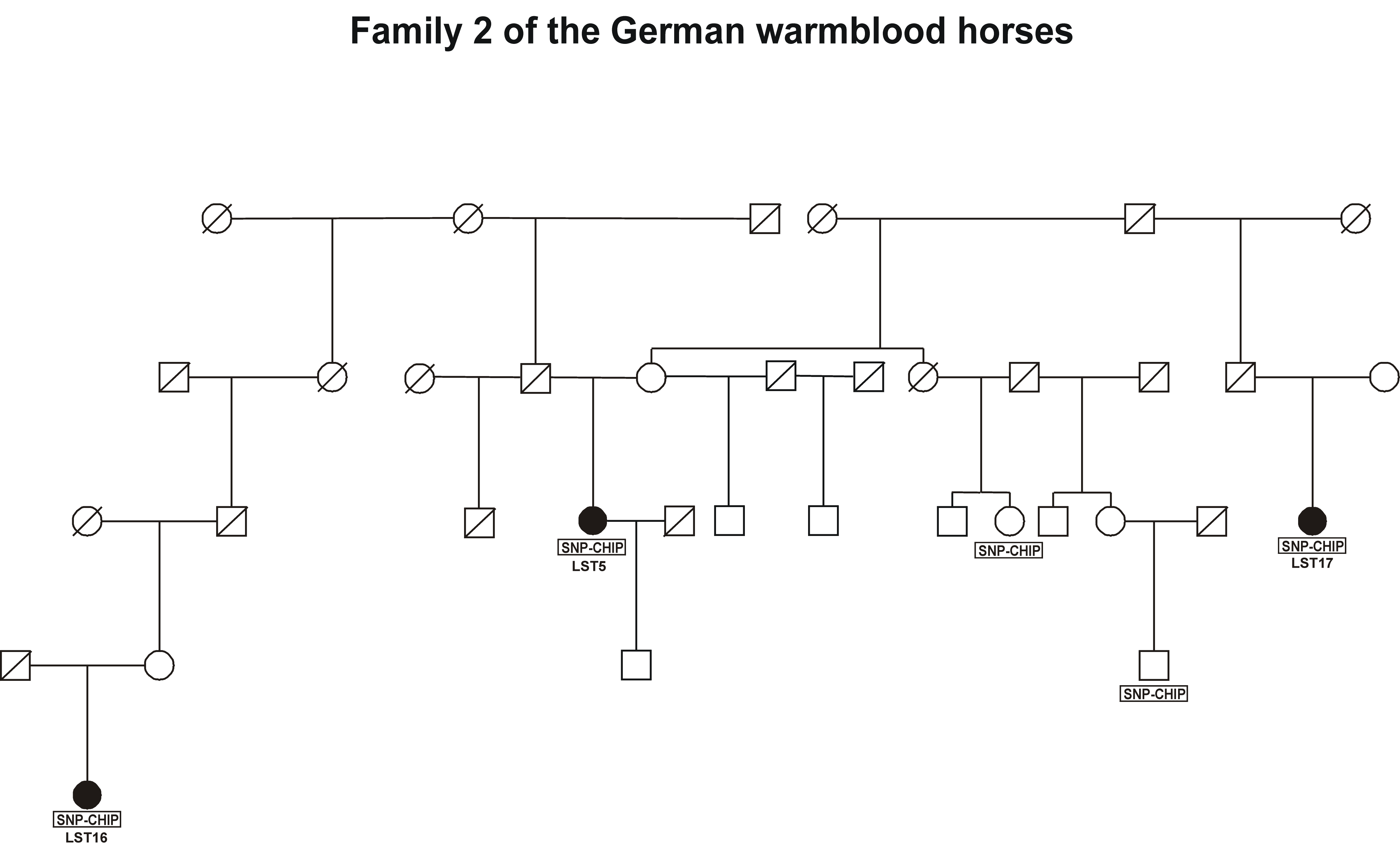


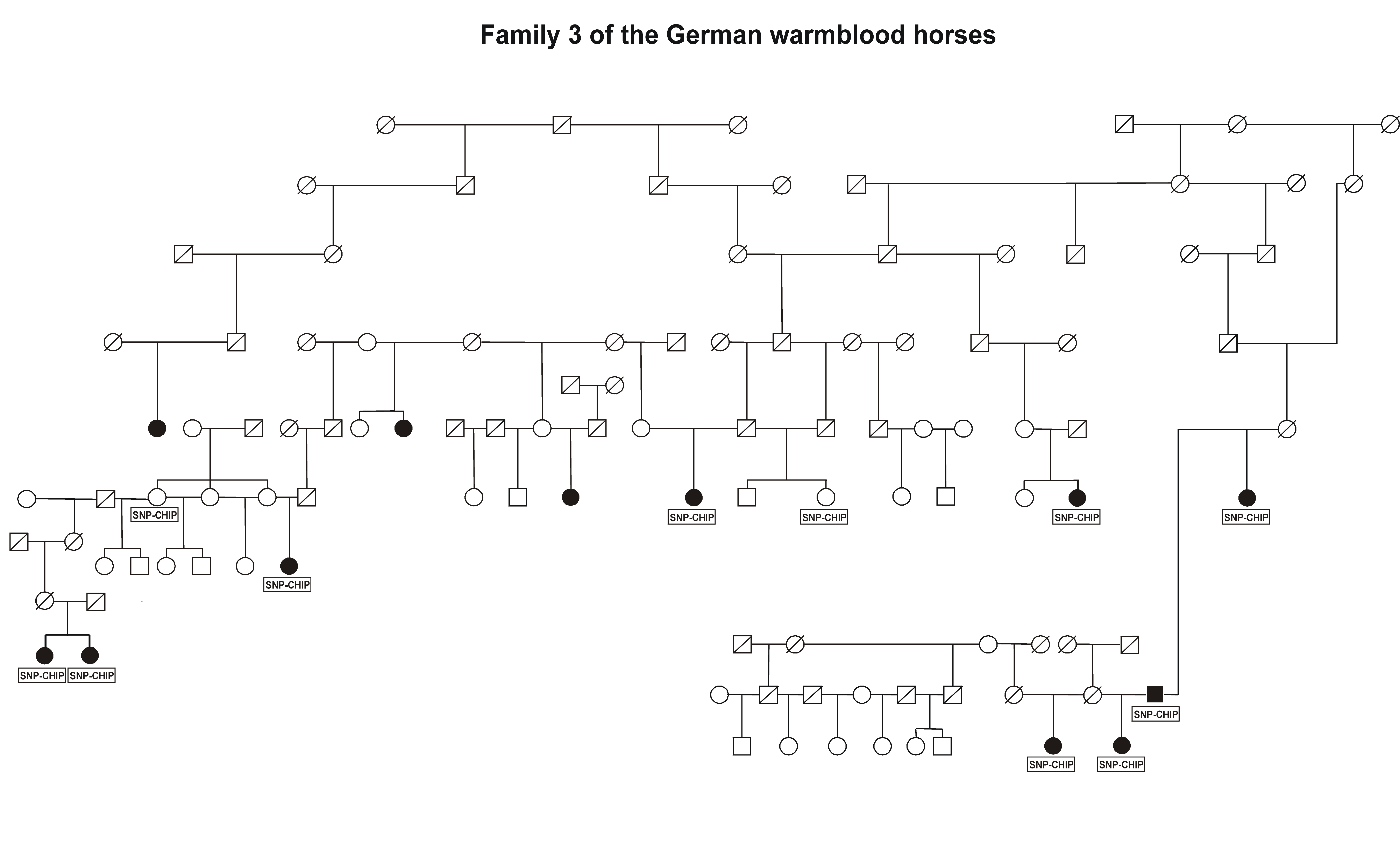


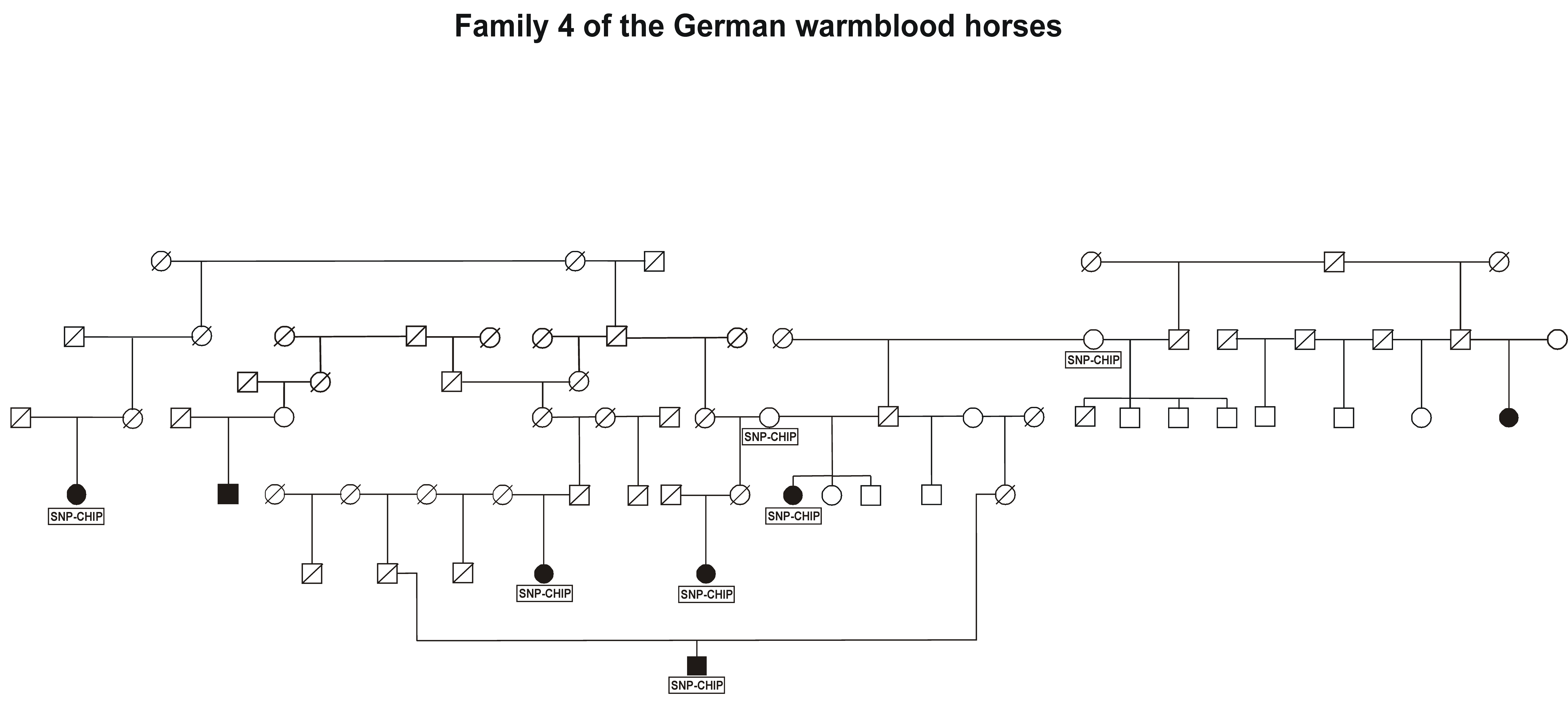


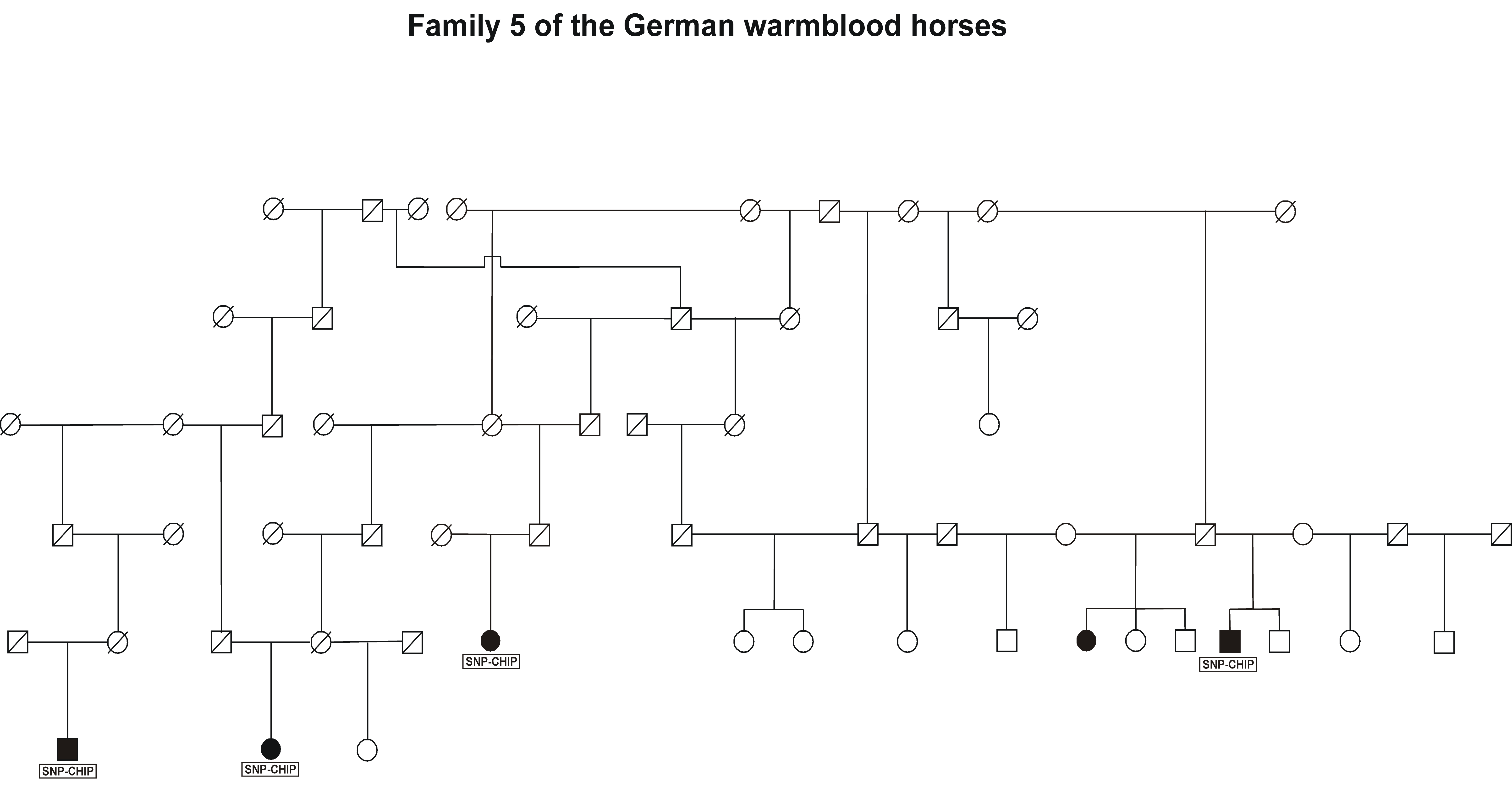

Supplement: Figure S7 — Pedigrees of the five Arabian and five German warmblood families used in multipoint linkage analyses. (DOC) [file pone.0041640.s007.doc]
