# Supplementary material for: Genome-Wide Linkage and Association Analysis Identifies Major Gene Loci for Guttural Pouch Tympany in Arabian and German Warmblood Horses
Source: PLoS One. 2012 Jul 27;7(7):e41640. doi: 10.1371/journal.pone.0041640 (PMC3407181; doi:10.1371/journal.pone.0041640)
Supplement: Table S1 — Results from the genome-wide linkage analysis (ECA15). Multipoint chromosome-wide significant Zmeans and LOD scores, their chromosome-wide P-values (Pz, PL) and positions in Mb for all Arabian horses. Genome-wide significant P-values<0.05 at 64–65 are in bold. (DOC) [file pone.0041640.s008.doc]

**Table S1.** **Results from the genome-wide linkage analysis (ECA15).** Multipoint chromosome-wide significant Zmeans and LOD scores, their chromosome-wide P-values (P_z_, P_L_) and positions in Mb for all Arabian horses. Genome-wide significant P-values <0.05 at 64-65 are in bold.

| ECA | Position (Mb) | Zmean | P_Z_ | LOD score | P_L_ |
| --- | --- | --- | --- | --- | --- |
| 15 | 47,572,962 | 3.08 | 0.001 | 1.49 | 0.004 |
| 15 | 48,050,187 | 3.11 | 0.0009 | 1.51 | 0.004 |
| 15 | 48,501,888 | 3.14 | 0.0008 | 1.53 | 0.004 |
| 15 | 49,189,435 | 3.18 | 0.0007 | 1.55 | 0.004 |
| 15 | 49,690,601 | 3.20 | 0.0007 | 1.56 | 0.004 |
| 15 | 50,058,011 | 3.22 | 0.0006 | 1.57 | 0.004 |
| 15 | 50,570,268 | 3.23 | 0.0006 | 1.58 | 0.004 |
| 15 | 51,041,508 | 3.23 | 0.0006 | 1.58 | 0.003 |
| 15 | 51,596,762 | 3.24 | 0.0006 | 1.58 | 0.003 |
| 15 | 52,085,272 | 3.23 | 0.0006 | 1.58 | 0.004 |
| 15 | 52,520,450 | 3.23 | 0.0006 | 1.58 | 0.004 |
| 15 | 53,093,079 | 3.22 | 0.0006 | 1.58 | 0.004 |
| 15 | 53,530,208 | 3.21 | 0.0007 | 1.57 | 0.004 |
| 15 | 54,014,468 | 3.22 | 0.0006 | 1.58 | 0.003 |
| 15 | 55,230,333 | 3.25 | 0.0006 | 1.60 | 0.003 |
| 15 | 57,014,242 | 3.15 | 0.0008 | 1.50 | 0.004 |
| 15 | 59,435,327 | 3.32 | 0.0004 | 1.58 | 0.004 |
| 15 | 60,007,477 | 3.45 | 0.0003 | 1.64 | 0.003 |
| 15 | 60,823,225 | 3.66 | 0.00013 | 1.72 | 0.002 |
| 15 | 61,044,054 | 3.69 | 0.00011 | 1.72 | 0.002 |
| 15 | 61,576,938 | 3.75 | 0.00009 | 1.72 | 0.002 |
| 15 | 62,041,648 | 3.80 | 0.00007 | 1.70 | 0.003 |
| 15 | 62,526,687 | 3.81 | 0.00007 | 1.66 | 0.003 |
| 15 | 63,007,330 | 3.98 | 0.00003 | 1.81 | 0.002 |
| 15 | 63,535,659 | 4.10 | 0.00002 | 1.90 | 0.002 |
| 15 | **64,087,624** | **4.14** | **0.00002** | **1.93** | **0.0014** |
| 15 | **64,551,633** | **4.16** | **0.00002** | **1.94** | **0.0014** |
| 15 | **65,298,924** | **4.12** | **0.00002** | **1.94** | **0.0014** |
| 15 | **65,519,182** | **4.08** | **0.00002** | **1.94** | **0.0014** |
| 15 | 66,019,470 | 3.92 | 0.00004 | 1.90 | 0.002 |
| 15 | 66,547,035 | 3.6 | 0.0002 | 1.82 | 0.002 |

**Table S1** **continued.**

| ECA | Position (Mb) | Zmean | P_Z_ | LOD score | P_L_ |
| --- | --- | --- | --- | --- | --- |
| 15 | 67,012,855 | 3.30 | 0.0005 | 1.74 | 0.002 |
| 15 | 69,087,991 | 1.98 | 0.02 | 1.29 | 0.007 |
| 15 | 71,041,540 | 2.97 | 0.0015 | 1.48 | 0.004 |
| 15 | 71,588,357 | 3.05 | 0.0011 | 1.48 | 0.004 |
| 15 | 72,204,285 | 3.12 | 0.0009 | 1.48 | 0.004 |
| 15 | 72,505,397 | 3.15 | 0.0008 | 1.48 | 0.004 |
| 15 | 73,009,720 | 3.17 | 0.0008 | 1.48 | 0.005 |
| 15 | 73,565,731 | 3.18 | 0.0007 | 1.47 | 0.005 |
| 15 | 74,039,700 | 3.18 | 0.0007 | 1.46 | 0.005 |
| 15 | 74,510,967 | 3.17 | 0.0008 | 1.45 | 0.005 |
| 15 | 75,204,646 | 3.15 | 0.0008 | 1.43 | 0.005 |
| 15 | 75,603,980 | 3.15 | 0.0008 | 1.42 | 0.005 |
| 15 | 76,004,165 | 3.15 | 0.0008 | 1.42 | 0.005 |
| 15 | 76,500,627 | 3.15 | 0.0008 | 1.42 | 0.005 |
| 15 | 77,114,780 | 3.22 | 0.0006 | 1.50 | 0.004 |
| 15 | 77,523,699 | 3.24 | 0.0006 | 1.51 | 0.004 |
| 15 | 78,013,519 | 3.25 | 0.0006 | 1.53 | 0.004 |
| 15 | 78,542,170 | 3.24 | 0.0006 | 1.53 | 0.004 |
| 15 | 79,016,728 | 3.22 | 0.0006 | 1.52 | 0.004 |
| 15 | 79,538,919 | 3.19 | 0.0007 | 1.50 | 0.004 |
| 15 | 80,085,648 | 3.16 | 0.0008 | 1.48 | 0.005 |
| 15 | 80,654,797 | 3.13 | 0.0009 | 1.46 | 0.005 |
| 15 | 81,143,358 | 3.09 | 0.001 | 1.44 | 0.005 |
| 15 | 81,524,261 | 3.04 | 0.0012 | 1.42 | 0.005 |
| 15 | 67,012,855 | 3.30 | 0.0005 | 1.74 | 0.002 |
| 15 | 69,087,991 | 1.98 | 0.02 | 1.29 | 0.007 |
| 15 | 71,041,540 | 2.97 | 0.0015 | 1.48 | 0.004 |
| 15 | 71,588,357 | 3.05 | 0.0011 | 1.48 | 0.004 |
| 15 | 72,204,285 | 3.12 | 0.0009 | 1.48 | 0.004 |
| 15 | 72,505,397 | 3.15 | 0.0008 | 1.48 | 0.004 |
| 15 | 73,009,720 | 3.17 | 0.0008 | 1.48 | 0.005 |
| 15 | 73,565,731 | 3.18 | 0.0007 | 1.47 | 0.005 |
| 15 | 74,039,700 | 3.18 | 0.0007 | 1.46 | 0.005 |
| 15 | 74,510,967 | 3.17 | 0.0008 | 1.45 | 0.005 |
| 15 | 75,204,646 | 3.15 | 0.0008 | 1.43 | 0.005 |
| 15 | 75,603,980 | 3.15 | 0.0008 | 1.42 | 0.005 |
| 15 | 76,004,165 | 3.15 | 0.0008 | 1.42 | 0.005 |
| 15 | 76,500,627 | 3.15 | 0.0008 | 1.42 | 0.005 |
| 15 | 77,114,780 | 3.22 | 0.0006 | 1.50 | 0.004 |

**Table S1** continued.

| ECA | Position (Mb) | Zmean | P_Z_ | LOD score | P_L_ |
| --- | --- | --- | --- | --- | --- |
| 15 | 77,523,699 | 3.24 | 0.0006 | 1.51 | 0.004 |
| 15 | 78,013,519 | 3.25 | 0.0006 | 1.53 | 0.004 |
| 15 | 78,542,170 | 3.24 | 0.0006 | 1.53 | 0.004 |
| 15 | 79,016,728 | 3.22 | 0.0006 | 1.52 | 0.004 |
| 15 | 79,538,919 | 3.19 | 0.0007 | 1.50 | 0.004 |
| 15 | 80,085,648 | 3.16 | 0.0008 | 1.48 | 0.005 |
| 15 | 80,654,797 | 3.13 | 0.0009 | 1.46 | 0.005 |
| 15 | 81,143,358 | 3.09 | 0.001 | 1.44 | 0.005 |
| 15 | 81,524,261 | 3.04 | 0.0012 | 1.42 | 0.005 |
