# Supplementary material for: Genome-Wide Linkage and Association Analysis Identifies Major Gene Loci for Guttural Pouch Tympany in Arabian and German Warmblood Horses
Source: PLoS One. 2012 Jul 27;7(7):e41640. doi: 10.1371/journal.pone.0041640 (PMC3407181; doi:10.1371/journal.pone.0041640)
Supplement: Table S2 — Results from the genome-wide linkage analysis (ECA3). Multipoint chromosome-wide significant Zmeans and LOD scores, their chromosome-wide P-values (Pz, PL) and positions in Mb for all German warmblood horses. Genome-wide significant P-values<0.05 at 34–55 Mb are in bold. (DOC) [file pone.0041640.s009.doc]

**Table S2**. **Results from the genome-wide linkage analysis (ECA3).** Multipoint chromosome-wide significant Zmeans and LOD scores, their chromosome-wide P-values (P_z_, P_L_) and positions in Mb for all German warmblood horses. Genome-wide significant P-values <0.05 at 34-55 Mb are in bold.

| ECA | Position (Mb) | Zmean | P_Z_ | LOD score | P_L_ |
| --- | --- | --- | --- | --- | --- |
| 3 | 33,216,203 | 2.11 | 0.02 | 1.24 | 0.008 |
| 3 | 33,386,111 | 2.16 | 0.02 | 1.28 | 0.008 |
| 3 | 33,513,009 | 2.19 | 0.014 | 1.30 | 0.007 |
| 3 | 33,695,098 | 2.23 | 0.013 | 1.33 | 0.007 |
| 3 | 33,788,850 | 2.26 | 0.012 | 1.35 | 0.006 |
| 3 | 33,889,026 | 2.30 | 0.011 | 1.38 | 0.006 |
| 3 | 34,005,755 | 2.47 | 0.007 | 1.48 | 0.005 |
| 3 | 34,234,892 | 2.92 | 0.002 | 1.68 | 0.003 |
| 3 | **34,443,396** | **3.24** | **0.0006** | **1.79** | **0.002** |
| 3 | **34,502,128** | **3.33** | **0.0004** | **1.82** | **0.002** |
| 3 | **34,673,405** | **3.53** | **0.0002** | **1.88** | **0.002** |
| 3 | **34,786,570** | **3.59** | **0.0002** | **1.90** | **0.002** |
| 3 | **35,140,330** | **3.78** | **0.00008** | **1.96** | **0.0013** |
| 3 | **35,279,221** | **3.88** | **0.00005** | **1.99** | **0.0012** |
| 3 | **35,408,432** | **3.94** | **0.00004** | **2.01** | **0.0012** |
| 3 | **35,502,786** | **4.01** | **0.00003** | **2.03** | **0.0011** |
| 3 | **35,639,914** | **4.06** | **0.00002** | **2.05** | **0.0011** |
| 3 | **36,461,592** | **4.32** | **0.00001** | **2.12** | **0.0009** |
| 3 | **36,840,265** | **4.42** | **0.00001** | **2.15** | **0.0008** |
| 3 | **36,977,339** | **4.44** | **4.50E-06** | **2.16** | **0.0008** |
| 3 | **37,121,854** | **4.46** | **4.10E-06** | **2.17** | **0.0008** |
| 3 | **37,148,447** | **4.47** | **3.91E-06** | **2.17** | **0.0008** |
| 3 | **37,398,507** | **4.52** | **3.09E-06** | **2.19** | **0.0008** |
| 3 | **37,540,091** | **4.55** | **2.68E-06** | **2.20** | **0.0007** |
| 3 | **37,635,869** | **4.57** | **2.44E-06** | **2.20** | **0.0007** |
| 3 | **37,789,700** | **4.60** | **2.11E-06** | **2.21** | **0.0007** |
| 3 | **37,957,578** | **4.63** | **1.83E-06** | **2.23** | **0.0007** |
| 3 | **38,123,494** | **4.67** | **1.51E-06** | **2.24** | **0.0007** |
| 3 | **38,157,802** | **4.68** | **1.43E-06** | **2.24** | **0.0007** |
| 3 | **38,344,351** | **4.72** | **1.18E-06** | **2.25** | **0.0006** |
| 3 | **38,417,961** | **4.74** | **1.07E-06** | **2.26** | **0.0006** |
| 3 | **38,502,764** | **4.76** | **9.68E-07** | **2.26** | **0.0006** |
| 3 | **38,637,583** | **4.80** | **7.93E-07** | **2.26** | **0.0006** |
| 3 | **38,767,613** | **4.86** | **5.87E-07** | **2.25** | **0.0006** |
| 3 | **38,930,295** | **4.96** | **3.52E-07** | **2.26** | **0.0006** |

Table S2 continued.

| ECA | Position (Mb) | Zmean | P_Z_ | LOD score | P_L_ |
| --- | --- | --- | --- | --- | --- |
| 3 | **39,054,277** | **5.01** | **2.72E-07** | **2.24** | **0.0007** |
| 3 | **39,250,079** | **5.09** | **1.79E-07** | **2.19** | **0.0008** |
| 3 | **39,375,909** | **5.20** | **9.96E-08** | **2.14** | **0.0008** |
| 3 | **39,547,631** | **5.36** | **4.16E-08** | **2.11** | **0.0009** |
| 3 | **39,642,405** | **5.45** | **2.52E-08** | **2.10** | **0.0009** |
| 3 | **39,861,536** | **5.77** | **3.96E-09** | **2.10** | **0.0009** |
| 3 | **40,179,308** | **6.54** | **3.08E-11** | **2.23** | **0.0007** |
| 3 | **40,523,770** | **7.28** | **1.67E-13** | **2.38** | **0.0005** |
| 3 | **40,650,253** | **7.54** | **2.35E-14** | **2.47** | **0.0004** |
| 3 | **40,781,763** | **7.88** | **1.64E-15** | **2.66** | **0.0002** |
| 3 | **41,027,613** | **8.32** | **4.40E-17** | **2.83** | **0.0002** |
| 3 | **41,230,999** | **8.57** | **5.17E-18** | **2.90** | **0.00013** |
| 3 | **41,368,801** | **8.65** | **2.57E-18** | **2.92** | **0.00012** |
| 3 | **41,424,399** | **8.67** | **2.16E-18** | **2.92** | **0.00012** |
| 3 | **41,567,820** | **8.71** | **1.52E-18** | **2.92** | **0.00012** |
| 3 | **41,631,052** | **8.72** | **1.39E-18** | **2.92** | **0.00012** |
| 3 | **41,842,702** | **8.75** | **1.07E-18** | **2.92** | **0.00012** |
| 3 | **41,928,376** | **8.75** | **1.07E-18** | **2.92** | **0.00012** |
| 3 | **42,002,976** | **8.75** | **1.07E-18** | **2.91** | **0.00012** |
| 3 | **42,181,036** | **8.76** | **9.76E-19** | **2.91** | **0.00013** |
| 3 | **42,286,717** | **8.76** | **9.76E-19** | **2.91** | **0.00013** |
| 3 | **42,411,355** | **8.76** | **9.76E-19** | **2.91** | **0.00013** |
| 3 | **42,654,399** | **8.77** | **8.93E-19** | **2.91** | **0.00013** |
| 3 | **42,765,698** | **8.76** | **9.76E-19** | **2.90** | **0.00013** |
| 3 | **42,946,562** | **8.75** | **1.07E-18** | **2.90** | **0.00013** |
| 3 | **43,022,497** | **8.75** | **1.07E-18** | **2.89** | **0.00013** |
| 3 | **43,140,945** | **8.73** | **1.27E-18** | **2.89** | **0.00013** |
| 3 | **43,265,818** | **8.70** | **1.66E-18** | **2.88** | **0.00013** |
| 3 | **43,419,199** | **8.66** | **2.36E-18** | **2.87** | **0.00014** |
| 3 | **43,519,205** | **8.64** | **2.81E-18** | **2.86** | **0.00014** |
| 3 | **43,772,912** | **8.56** | **5.64E-18** | **2.84** | **0.00015** |
| 3 | **43,927,551** | **8.54** | **6.71E-18** | **2.84** | **0.0002** |
| 3 | **44,037,117** | **8.51** | **8.70E-18** | **2.83** | **0.0002** |
| 3 | **44,250,904** | **8.44** | **1.59E-17** | **2.82** | **0.0002** |
| 3 | **44,525,939** | **8.32** | **4.40E-17** | **2.80** | **0.0002** |
| 3 | **44,630,433** | **8.26** | **7.28E-17** | **2.79** | **0.0002** |
| 3 | **44,944,877** | **7.95** | **9.33E-16** | **2.74** | **0.0002** |
| 3 | **45,003,697** | **7.86** | **1.92E-15** | **2.72** | **0.0002** |
| 3 | **45,228,417** | **7.46** | **4.33E-14** | **2.65** | **0.0002** |

Table S2 continued.

| ECA | Position (Mb) | Zmean | P_Z_ | LOD score | P_L_ |
| --- | --- | --- | --- | --- | --- |
| 3 | **45,259,290** | **7.38** | **7.91E-14** | **2.64** | **0.0002** |
| 3 | **45,449,463** | **6.75** | **7.39E-12** | **2.54** | **0.0003** |
| 3 | **45,688,039** | **6.27** | **1.81E-10** | **2.47** | **0.0004** |
| 3 | **45,882,542** | **5.88** | **2.05E-09** | **2.39** | **0.0004** |
| 3 | **46,087,924** | **5.48** | **2.13E-08** | **2.31** | **0.0006** |
| 3 | **46,381,805** | **5.03** | **2.45E-07** | **2.21** | **0.0007** |
| 3 | **46,513,178** | **4.86** | **5.87E-07** | **2.16** | **0.0008** |
| 3 | **46,668,091** | **4.65** | **1.66E-06** | **2.09** | **0.001** |
| 3 | **46,754,866** | **4.54** | **2.81E-06** | **2.07** | **0.001** |
| 3 | **46,875,647** | **4.36** | **0.00001** | **2.06** | **0.001** |
| 3 | **47,006,528** | **4.18** | **0.00001** | **2.05** | **0.0011** |
| 3 | **47,219,906** | **3.82** | **0.00007** | **2.01** | **0.0012** |
| 3 | **47,267,059** | **3.76** | **0.00009** | **2.00** | **0.0012** |
| 3 | **47,452,823** | **3.54** | **0.0002** | **1.96** | **0.0013** |
| 3 | **47,573,471** | **3.41** | **0.0003** | **1.94** | **0.0014** |
| 3 | **47,684,860** | **3.30** | **0.0005** | **1.90** | **0.002** |
| 3 | **47,766,129** | **3.26** | **0.0006** | **1.89** | **0.002** |
| 3 | **47,915,872** | **3.15** | **0.0008** | **1.86** | **0.002** |
| 3 | **48,072,006** | **3.77** | **0.00008** | **2.16** | **0.0008** |
| 3 | **48,307,263** | **3.88** | **0.00005** | **2.18** | **0.0008** |
| 3 | **48,375,438** | **3.86** | **0.00006** | **2.16** | **0.0008** |
| 3 | **48,519,426** | **3.76** | **0.00008** | **2.11** | **0.0009** |
| 3 | **48,726,615** | **3.64** | **0.00013** | **2.05** | **0.0011** |
| 3 | **48,955,067** | **3.67** | **0.00012** | **2.08** | **0.001** |
| 3 | **49,031,634** | **3.68** | **0.00012** | **2.09** | **0.001** |
| 3 | **49,169,369** | **3.68** | **0.00012** | **2.10** | **0.0009** |
| 3 | **49,476,446** | **3.69** | **0.00011** | **2.12** | **0.0009** |
| 3 | **49,604,491** | **3.71** | **0.00011** | **2.14** | **0.0009** |
| 3 | **49,680,458** | **3.71** | **0.0001** | **2.15** | **0.0008** |
| 3 | **49,923,098** | **3.74** | **0.00009** | **2.17** | **0.0008** |
| 3 | **50,059,970** | **3.77** | **0.00008** | **2.19** | **0.0007** |
| 3 | **50,135,295** | **3.77** | **0.00008** | **2.20** | **0.0007** |
| 3 | **50,328,540** | **3.79** | **0.00008** | **2.21** | **0.0007** |
| 3 | **50,444,836** | **3.80** | **0.00007** | **2.22** | **0.0007** |
| 3 | **50,561,890** | **3.79** | **0.00008** | **2.22** | **0.0007** |
| 3 | **50,687,481** | **3.78** | **0.00008** | **2.22** | **0.0007** |
| 3 | **50,757,887** | **3.76** | **0.00008** | **2.22** | **0.0007** |
| 3 | **50,953,486** | **3.73** | **0.0001** | **2.21** | **0.0007** |
| 3 | **51,128,517** | **3.70** | **0.00011** | **2.20** | **0.0007** |

**Table S2** continued.

| ECA | Position (Mb) | Zmean | P_Z_ | LOD score | P_L_ |
| --- | --- | --- | --- | --- | --- |
| 3 | **51,261,819** | **3.67** | **0.00012** | **2.17** | **0.0008** |
| 3 | **51,525,184** | **3.59** | **0.0002** | **2.14** | **0.0008** |
| 3 | **51,729,578** | **3.49** | **0.0002** | **2.13** | **0.0009** |
| 3 | **51,782,286** | **3.46** | **0.0003** | **2.11** | **0.0009** |
| 3 | **51,894,540** | **3.40** | **0.0003** | **2.07** | **0.001** |
| 3 | **52,033,022** | **3.32** | **0.0004** | **2.05** | **0.0011** |
| 3 | **52,129,820** | **3.27** | **0.0005** | **1.99** | **0.0012** |
| 3 | **52,272,374** | **3.13** | **0.0009** | **1.91** | **0.002** |
| 3 | **52,442,712** | **2.95** | **0.002** | **1.84** | **0.002** |
| 3 | 52,577,112 | 2.80 | 0.003 | 1.81 | 0.002 |
| 3 | 52,638,506 | 2.76 | 0.003 | 1.73 | 0.002 |
| 3 | 52,818,226 | 2.61 | 0.005 | 1.62 | 0.003 |
| 3 | 53,026,058 | 2.43 | 0.008 | 1.44 | 0.005 |
| 3 | 53,312,993 | 2.10 | 0.02 | 1.36 | 0.006 |
| 3 | 53,396,596 | 1.98 | 0.02 | 1.70 | 0.003 |
| 3 | 53,842,980 | 2.76 | 0.003 | 1.75 | 0.002 |
| 3 | **53,943,527** | **2.95** | **0.002** | **1.81** | **0.002** |
| 3 | **54,082,267** | **3.22** | **0.0006** | **1.84** | **0.002** |
| 3 | **54,170,688** | **3.38** | **0.0004** | **1.86** | **0.002** |
| 3 | **54,268,493** | **3.53** | **0.0002** | **1.88** | **0.002** |
| 3 | **54,400,643** | **3.67** | **0.00012** | **1.89** | **0.002** |
| 3 | **54,588,816** | **3.85** | **0.00006** | **1.88** | **0.002** |
| 3 | **54,796,404** | **3.94** | **0.00004** | **1.85** | **0.002** |
| 3 | **54,993,365** | **3.97** | **0.00004** | **1.84** | **0.002** |
| 3 | **55,007,716** | **3.97** | **0.00004** | **1.81** | **0.002** |
| 3 | **55,141,235** | **3.94** | **0.00004** | **1.77** | **0.002** |
| 3 | **55,323,036** | **3.88** | **0.00005** | **1.74** | **0.002** |
| 3 | **55,395,226** | **3.84** | **0.00006** | **1.74** | **0.002** |
| 3 | **55,713,990** | **3.64** | **0.00014** | **1.73** | **0.002** |
| 3 | **55,940,167** | **3.50** | **0.0002** | **1.72** | **0.002** |
| 3 | 56,044,340 | 3.40 | 0.0003 | 1.66 | 0.003 |
| 3 | 56,208,321 | 3.20 | 0.0007 | 1.64 | 0.003 |
| 3 | 56,270,771 | 3.14 | 0.0009 | 1.58 | 0.003 |
| 3 | 56,380,863 | 3.00 | 0.0014 | 1.43 | 0.005 |
| 3 | 56,626,763 | 2.71 | 0.003 | 1.35 | 0.006 |
| 3 | 56,781,589 | 2.65 | 0.004 | 1.22 | 0.009 |
| 3 | 57,035,727 | 2.55 | 0.005 | 1.15 | 0.011 |
| 3 | 57,149,814 | 2.50 | 0.006 | 0.96 | 0.02 |
| 3 | 57,373,477 | 2.39 | 0.008 | 0.88 | 0.02 |

**Table S2** continued.

| ECA | Position (Mb) | Zmean | P_Z_ | LOD score | P_L_ |
| --- | --- | --- | --- | --- | --- |
| 3 | 57,557,254 | 2.34 | 0.01 | 0.88 | 0.02 |
| 3 | 57,658,626 | 2.30 | 0.011 | 0.80 | 0.03 |
| 3 | 57,876,814 | 2.29 | 0.011 | 0.78 | 0.03 |
| 3 | 58,026,750 | 2.27 | 0.012 | 0.76 | 0.03 |
| 3 | 58,136,031 | 2.26 | 0.012 | 0.74 | 0.03 |
| 3 | 58,250,695 | 2.24 | 0.012 | 0.73 | 0.03 |
| 3 | 58,378,586 | 2.23 | 0.013 | 0.73 | 0.03 |
| 3 | 58,669,130 | 2.21 | 0.014 | 0.72 | 0.03 |
| 3 | 58,832,110 | 2.18 | 0.015 | 0.71 | 0.04 |
| 3 | 58,894,601 | 2.15 | 0.02 | 0.71 | 0.04 |
