# Supplementary material for: Genome-Wide Linkage and Association Analysis Identifies Major Gene Loci for Guttural Pouch Tympany in Arabian and German Warmblood Horses
Source: PLoS One. 2012 Jul 27;7(7):e41640. doi: 10.1371/journal.pone.0041640 (PMC3407181; doi:10.1371/journal.pone.0041640)
Supplement: Table S3 — Survey on all animals genotyped for the genome-wide association study and by foals affected by guttural pouch tympany and unaffected animals in total and by breed and sex. (DOC) [file pone.0041640.s010.doc]

**Table S3** **Survey on all animals genotyped for the genome-wide association study and by foals affected by guttural pouch tympany and unaffected animals in total and by breed and sex.**

| Breed | Group | Number of animals | Affected animals | Unaffected animals | Affected females | | Affected males |
| --- | --- | --- | --- | --- | --- | --- | --- |
| Arabian horses | Member of a family (1-5) | 56 | 32 | 24 | 19 | 13 | |
|  | Without family | 1 | 1 | 0 | 0 | 1 | |
|  | Unrelated controls | 28 | 0 | 28 | 0 | 0 | |
| German  warm-  blood | Member of a family (1-5) | 42 | 36 | 6 | 28 | 8 | |
|  | Without family | 1 | 1 | 0 | 1 | 0 | |
|  | Unrelated controls | 330 | 0 | 330 | 0 | 0 | |
| Total |  | 458 | 70 | 388 | 48 | 22 | |
