# Supplementary material for: Genome-Wide Linkage and Association Analysis Identifies Major Gene Loci for Guttural Pouch Tympany in Arabian and German Warmblood Horses
Source: PLoS One. 2012 Jul 27;7(7):e41640. doi: 10.1371/journal.pone.0041640 (PMC3407181; doi:10.1371/journal.pone.0041640)
Supplement: Table S4 — Arabian and German warmblood horses used for the multipoint linkage analysis. Number of animals used to construct pedigrees and number of genotyped samples using the Illumina equine SNP50 beadchip by families and their affection status, number of affected foals and their distribution by sex and family and in total. (DOC) [file pone.0041640.s011.doc]

**Table S4.** **Arabian and German warmblood horses used for the multipoint linkage analysis.** Number of animals used to construct pedigrees and number of genotyped samples using the Illumina equine SNP50 beadchip by families and their affection status, number of affected foals and their distribution by sex and family and in total.

| Breed | Family | | Number of animals (genotyped) | Affected animals (genotyped) | Unaffected animals (genotyped) | Affected females (genotyped) | Affected males (genotyped) |
| --- | --- | --- | --- | --- | --- | --- | --- |
| Arabian horses | | 1 | 67(13) | 13(8) | 54(5) | 10(6) | 3(2) |
|  |  | 2 | 56(10) | 9(5) | 47(5) | 7(4) | 2(1) |
|  |  | 3 | 77(13) | 17(10) | 60(3) | 11(5) | 6(5) |
|  |  | 4 | 40(17) | 8(7) | 32(10) | 5(3) | 5(4) |
|  |  | 5 | 10(3) | 2(2) | 8(1) | 1(1) | 1(1) |
|  |  | 1-5 | 250(56) | 49(32) | 201(24) | 34(19) | 17(13) |
| German  warmblood | | 1 | 110(16) | 19(15) | 91(1) | 13(11) | 6(4) |
|  |  | 2 | 27(5) | 3(3) | 24(2) | 3(3) | 0(0) |
|  |  | 3 | 72(11) | 9(9) | 63(2) | 8(8) | 1(1) |
|  |  | 4 | 46(6) | 7(5) | 39(1) | 5(4) | 2(1) |
|  |  | 5 | 27(4) | 5(4) | 22(0) | 3(2) | 2(2) |
|  |  | 1-5 | 282(42) | 43(36) | 239(6) | 31(28) | 11(8) |
| Total | | 12 | 532(98) | 92(68) | 440(30) | 65(47) | 26(21) |
